# Recent Advances in 2D Lateral Heterostructures

**DOI:** 10.1007/s40820-019-0276-y

**Published:** 2019-06-05

**Authors:** Jianwei Wang, Zhiqiang Li, Haiyuan Chen, Guangwei Deng, Xiaobin Niu

**Affiliations:** 1 School of Materials and Energy, University of Electronic Science and Technology of China, Chengdu, 610054 People’s Republic of China; 2 Institute of Fundamental and Frontier Sciences, University of Electronic Science and Technology of China, Chengdu, 610054 People’s Republic of China

**Keywords:** Two-dimensional lateral heterostructures, Homogeneous junction, Heterogeneous junction, Electronic and photoelectronic properties, Tunable mechanisms

## Abstract

Recent developments in synthesis and nanofabrication technologies offer the tantalizing prospect of realizing various applications from two-dimensional (2D) materials. A revolutionary development is to flexibly construct many different kinds of heterostructures with a diversity of 2D materials. These 2D heterostructures play an important role in semiconductor and condensed matter physics studies and are promising candidates for new device designs in the fields of integrated circuits and quantum sciences. Theoretical and experimental studies have focused on both vertical and lateral 2D heterostructures; the lateral heterostructures are considered to be easier for planner integration and exhibit unique electronic and photoelectronic properties. In this review, we give a summary of the properties of lateral heterostructures with homogeneous junction and heterogeneous junction, where the homogeneous junctions have the same host materials and the heterogeneous junctions are combined with different materials. Afterward, we discuss the applications and experimental synthesis of lateral 2D heterostructures. Moreover, a perspective on lateral 2D heterostructures is given at the end.

## Highlights

- The tunable mechanisms of lateral heterostructures on both homogeneous junctions and heterogeneous junctions are summarized.
- Electronic and photoelectronic devices with lateral heterostructures have been discussed.
- Different types of contacts of 2D lateral heterostructures are classified.
- Recent developments in synthesis and nanofabrication technologies of 2D lateral heterostructures are reviewed.

## Graphical abstract

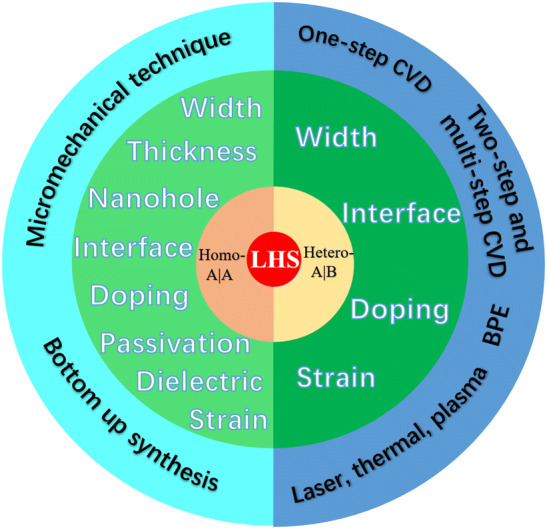

## Introduction

The graphene monolayer, which was successfully prepared through micromechanical cleavage in 2004, triggered enormous research interests because of its exotic physical properties, such as high charge carrier mobility, high mechanical strength, high thermal conductivity, and broadband optical absorption [1–4]. More and more researchers pay attention to studies of monolayer materials with honeycomb structures as interesting as graphene [5, 6], which give birth to a new research field, i.e., two-dimensional (2D) materials. Because of their uncommon properties and related potential applications, other 2D materials beyond graphene, including hexagonal boron nitride (h-BN) [7, 8], transition metal dichalcogenides (TMDCs) [9, 10], black phosphorus (BP) [11, 12], and silicone [13], are extensively investigated.

Other than monolayer 2D materials, 2D heterostructures have been investigated theoretically and experimentally, and their new properties are tailored, which provide many potential applications. Vertical 2D heterostructures composed of different single layers show some novel electronic [14] and optical properties [15], such as high carrier mobility [16] and perfect photoresponse performance [17], which can be used to design transistors [18, 19] and photoelectronic devices [20]. Different from vertical stacking structures, the lateral stitching structures in monolayer plane are also utilized to build functional devices. Many devices fabricated with lateral heterostructures (LHSs) have demonstrated superior performance or shown unique properties. Based on monolayer WSe_2_–WS_2_ heterojunctions, high-mobility field-effect transistors (FETs), *p*–*n* diodes with large rectification behavior, superior photovoltaic devices (large open-circuit voltage of 0.47 V and short-circuit current of 1.2 nA), and CMOS inverter with large current gain (as large as 24) have been demonstrated [21]. It has been shown that a photodetector based on MoS_2_–MoSe_2_ LHSs demonstrated an optimal photoresponse performance. The responsivity, detectivity, and external quantum efficiency reach 1.3 A W^−1^, 2.6 × 10^11^ Jones, and 263.1%, respectively [22]. In 2D bipolar transistors based on lateral WSe_2_–MoS_2_ heterojunctions, common emitter current gain and negative differential resistance (NDR) phenomenon were observed [23]. A light-emitting device designed with lateral WSe_2_–MoS_2_ heterostructure exhibited a larger conversion efficiency of 1 m% (the ratio of the emitted photon to the injected carriers) in 70 kw cm^−2^ than a homojunction device based on monolayer MoS_2_ [24]. Furthermore, photodetectors built with lateral heterojunction graphene and thin amorphous carbon films have a high photodetectivity of 10^13^ Jones and short response time of sub-100 µs [25]. These devices built with LHSs can potentially be used in future electronic circuits and device applications.

To realize more controllable device functions, how to tune the properties by structure has become a main issue of researchers’ concerns. The tunability of heterostructures based on 2D materials has been demonstrated in graphene-based heterostructures [26]. For example, stack sequences, doping, and different geometries have been proved as effective ways to modify the properties. Up to now, a large number of 2D heterojunctions fabricated in the laboratory can be identified mainly into two different types according to their structures [27–29]: (1) vertical heterostructures stacked layer by layer; (2) LHSs where the 2D materials are stitched seamlessly in a panel. In vertical 2D heterostructures, the isolated atomic component can be assembled to form new layered materials stacked in a precisely selected sequence. The different layers in vertical heterostructures are generally combined by van der Waals (vdW) interaction. In lateral 2D heterostructures, different 2D atomic panels are stitched in a single atomic layer because of the similar structure and small lattice mismatch. The chemical bonding between the edges of different panels plays an important role in the combination of two different 2D materials. In general, the vdW interaction is weaker than the chemical bonding. Because of the different combination strengths of the two types of heterostructures at the interface, the synthesis of them has distinct differences. Owing to vdW weak interaction, mechanical exfoliation and mechanical transfer techniques are successfully used in fabricating the vertical heterostructure, which makes them become some of the hottest research fields in recent years. However, there are two main issues which restrict the performance of 2D devices based on vertical heterostructures: (1) contaminants between layers and (2) stacking orientation induced in the stacking process [30, 31]. To overcome these limitations, lateral heterojunction was proposed as one of the solutions. Enhanced intrinsic performances compared with those of graphene-based vertical transistors have been reported in LHSs [32]. The LHSs are synthesized by direct growth, and the two panels can be stitched seamlessly, which forms a sharp interface. The quality and orientation of the inner interfaces in LHSs can be precisely controlled. The interface between the two in-plane panels can induce many attractive properties, which are related to the microstructure of the interface. LHSs usually offer larger tunability of band offset and can easily modify electronic properties. These advantages make it valuable to design potential function devices using LHSs.

There are some reviews talking about the synthesis of 2D materials and their heterostructures [33–35]. The chemical vapor deposition (CVD) method is the main synthesis method for LHSs, which is mostly the concern. However, some novel methods for synthesizing LHSs have been ignored. Here, the one-step, two-step, and multi-step growths and some other methods for the fabrication of LHSs are summarized. Although some summaries of synthesis methods used in 2D materials and their heterojunctions have already been given [33–36], little is known about the developments of LHSs. A summary of physical properties, tunable or modulation mechanisms, device applications, and synthesis of LHSs is desirable. We hope our summary can provide an insight on recent developments of LHSs and can inspire future studies on LHSs.

**Scheme 1:**
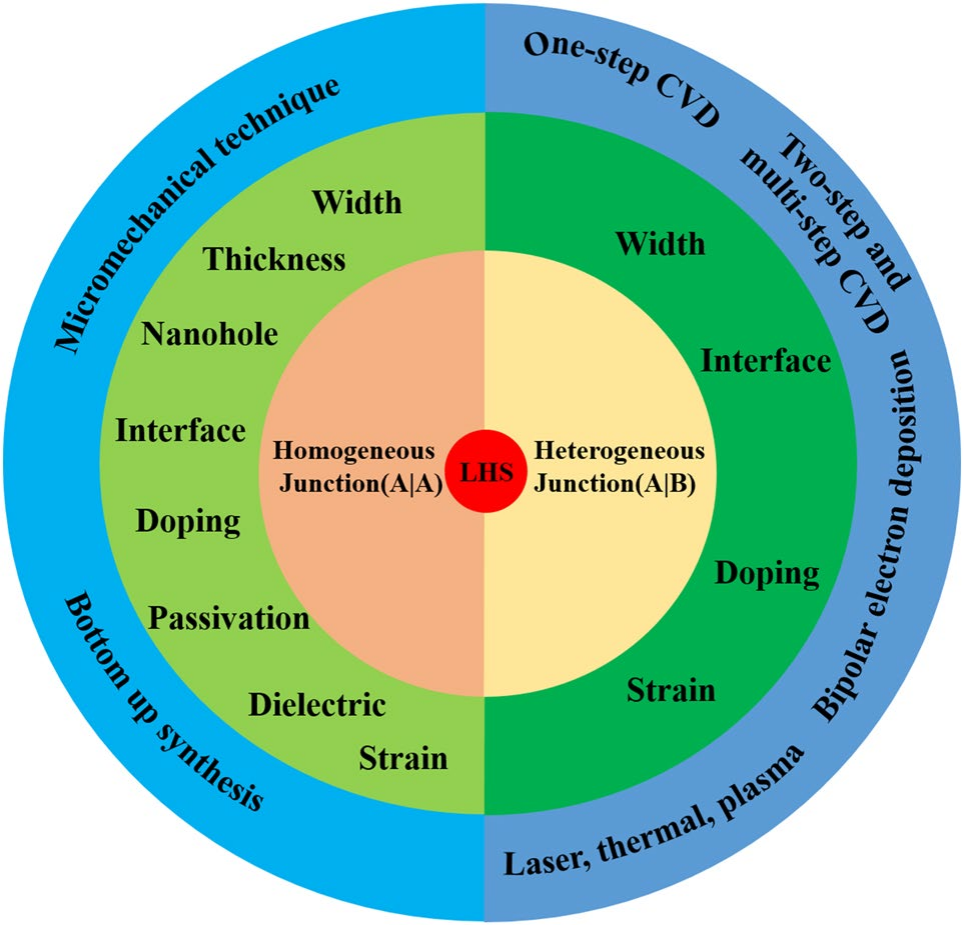
Tunable mechanism and synthesis method of the lateral heterostructures

In this review, we summarized the progresses on 2D LHSs properties, applications, and experimental synthesis. First, the properties of homogeneous and heterogeneous junction LHSs related to the structure (interface, width, nanohole, thickness, strain, and dielectric), doping, and passivation have been investigated. Second, electronic and optical device applications for lateral heterostructures have been summarized. Then, applications of LHSs in electronic and photoelectronic devices are given. In the fourth part, we talk about the synthesis status of LHSs. In the end, a short perspective on LHSs is given. In order to indicate the tunable properties and synthesis method simply, Scheme 1 is used to introduce the research status clearly.

## The Properties of LHSs

With developments on theory and experiment in 2D LHSs, more and more heterostructures have been designed and synthesized. The properties of LHSs are important for device application. In order to introduce important developments about recent works on LHSs, we divide them into two types, as given in Table 1. The LHSs can be classified into two types, according to the materials on the sides of the interface: (1) homogeneous junctions (*A*|*A*), where the host materials are the same on both sides of the interface, and (2) heterogeneous junctions (*A*|*B*), where the host materials are different.

**Table 1:** Strategies used in building lateral heterojunction

	Tenability	Example	Contact type	Refs.
Homogeneous junctions (*A*|*A*)	1. Structure: interface, width, nanohole and thickness	aGNR|zGNR	M/S	[41]
		(m)aGNR|(n)aGNR	Semi-M/S	[43]
		Black–blue phosphorene	S/S types I, II	[46]
		(m)GeP_3_|(n)GeP_3_	S/S type II	[47]
		GNM/graphene	S/Semi-M	[55]
		Monolayer–multilayer MoS_2_	S/S type I	[56]
		Multilayer MoSe_2_	S/S type II	[151]
	2. Doping and passivation: substituting doping, surface adsorption, monohydrogenated, dihydrogenated	*n*-doped/*p*-doped GNR	S/S type III	[63]
		*n*-doped GNR/GNR (*n*-doped GNR)	S/S type II	[131]
		H_2_-doped (m)zGNR–H	M/S	[75]
		O/zGNR–H/zGNR	M/S	[80]
		H_2_–(m)zGNR–H/H–(n)zGNR–H	M/S	[82]
		zMoS_2_NR–H/zMoS_2_NR	M/S	[85]
		H–6ZSiNR–H/H_2_–6ZSiNR–H_2_	M/S	[86]
	3. Strain and dielectric	Graphene	M/S	[91]
		ReSe_2_	S/S type I	[87]
		MoS_2_	S/S type I	[95]
Heterogeneous junctions (*A*|*B*)	1. Structure: interface and width	MoS_2_–(MX_2_)*n* (*M* = Mo, W, *X* = S/Se)	S/S type II	[108]
		Arsenene/blue phosphorene	S/S type II	[114]
		Graphene–h-BN	M/S	[116]
		Graphene-aPNR	M/S	[118]
		Graphene–TMDC	M/S	[119]
		h-BN–TMDC	S/S	[142]
	2. Doping and passivation: H doping	Graphene–aPNR (H-doped)		[118]
		Doped WSe_2_–WS_2_	S/S type II	[130]

*A* and *B* represent different materials. *M* and *S* represent the metal and semiconductor, respectively

### Homogeneous Junctions

Given the same materials across the junctions, many ways have been proposed to modulate the heterostructure. As we stated above, the doping and geometry structures provide the tunable mechanisms of the LHSs. Here, the variations of structure (interface, width, nanohole, and thickness), doping, passivation, strain, and dielectric have been used to tune the physical properties of host materials in the LHSs. We will discuss them, respectively.

#### Structure: Interface, Width, Nanohole, and Thickness

**Fig. 1:**
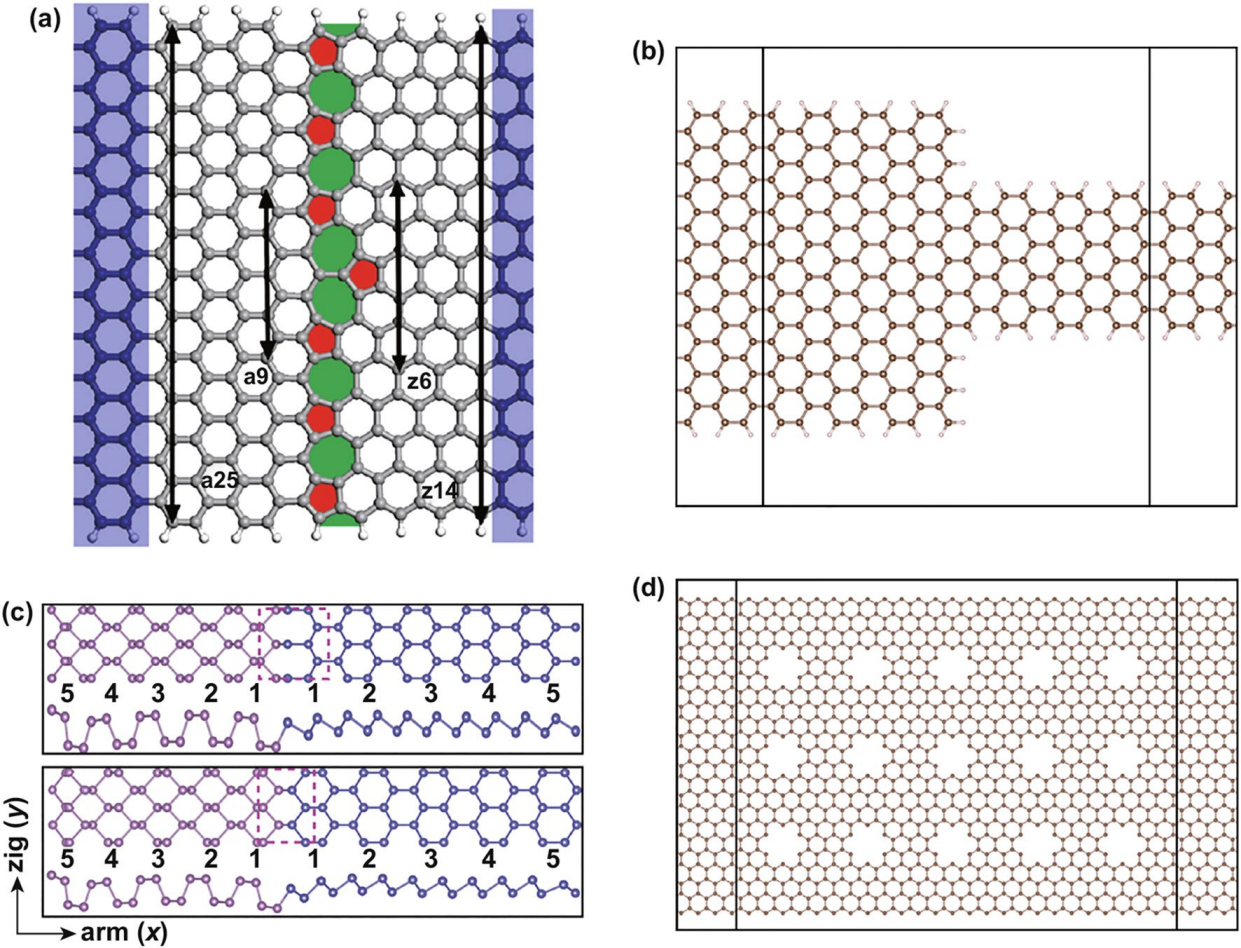
**a** Heterojunction of armchair25 (9)| zigzag14 (6) GNR. The interface is of pentagon–heptagon pairs, corresponding to red and green holes in the picture. Reprinted with permission from Ref. [41]. **b** Heterojunction of armchair20_armchair9 GNR; the edges are passivated with hydrogen atoms. **c** Top and side views of 5–5 black–blue phosphorene LHS with an octatomic-ring interface and a hexatomic-ring interface. The pink dashed line marks the interface region. Reprinted with permission from Ref. [46]. **d** Simple drawing of the GNM heterostructures with circle nanoholes. (Color figure online)

As we know, if graphene is cut along different directions (zigzag or armchair direction), different types of nanoribbon edges can be obtained. The zigzag graphene nanoribbons (zGNR) behaves as a metal at nonmagnetic state, and the armchair graphene nanoribbons (aGNR) behaves as a semiconductor. The energy gaps of armchair graphene nanoribbons oscillate with the ribbon width, which obeys the relation △3*p* + 1 ≥ △3*p* > △3*p* + 2 (*p* is an integer) [37, 38]. If the zigzag and armchair graphene nanoribbons are fused in an atomic layer, a semimetal and semiconductor junction can be formed. The different topological defects (the ring structures at the interface) can be found with different GNR units. It is shown that different ring structures at the interface can influence the transport properties and conductivity of the junctions. The most stable armchair–zigzag GNR junction is the 5–7–5-ring structure [39, 40]. Li et al. [41] designed a heterojunction combining a zGNR and an aGNR, which is shown in Fig. 1a. The structure has the defects of pentagon–heptagon pairs and clearly has rectification effect. The electronic properties of the heterojunction can be tuned by the width: When the width is 6*p* + 5 (*p* = 1, 2, …), it behaves as a metal; when the width is 6*p* + 1 or 6*p* + 3 (*p* = 1, 2, …), it behaves as a semiconductor. With different widths, the heterojunctions have oscillating conductance. The rectification properties mainly come from the destructive interferences. The rectification properties of this structure can also be tuned by the width of each of the components of the heterojunction. For this semiconducting junction, by changing the width of the zGNR, a large rectification ratio can be obtained, through reducing the backward current [41]. They also studied the graphene nanoribbon heterojunction, which was designed by combining an armchair graphene nanoribbon and a zigzag graphene nanoribbon side by side. By controlling the widths of the nanoribbons, the electronic properties of the heterojunctions can be tuned between metallic and semiconducting: When the width of armchair graphene nanoribbon is 3*p* + 2, the heterojunction behaves metallically; when the width is 3*p* or 3*p* + 1, it behaves as a semiconductor (*p* = 1, 2, 3, …). The rectification behaviors are influenced mainly by the side connection length between aGNR and zGNR. The different interface defects cause the asymmetrical transmission of electrons and holes, which induces the rectification [42]. In brief, the different edge connections can induce a different interface, which exhibits the powerful tunability of the electronic properties.

The electronic properties of 2D materials can be tuned by the ribbon width. The armchair graphene nanoribbons can behave as semiconductors, and the energy gaps can be tuned by the ribbon width, which makes it an appropriate component for heterojunction design. When the widths of the aGNRs are 3*p* and 3*p* + 1, the aGNRs behave as semiconductors, whose bandgaps range from 0.4 to 2.5 eV; when the width is 3*p* + 2, the aGNRs behaves as a semimetal, whose bandgaps range from 0 to 0.3 eV (*p* = 1, 2, 3, …). An undoped armchair graphene heterojunction has been proposed with left and right parts having different widths, where the left part is a semimetal and the right part is a semiconductor [43]. The heterojunction has rectification effect induced by the interface, which is similar to the Schottky barrier, and the rectification ratio is affected by the ribbon width (left width/right width). The decreasing width of the semiconducting part makes electron transport more difficult and induces a larger rectification ratio. In Fig. 1b, a similar armchair graphene nanoribbon heterojunction has been shown, which has different widths in the left (20) and right (9). In the same way, the cross-bar and *T*-shaped configured heterojunctions based on armchair graphene and barbell-shaped graphene nanoribbon heterojunctions have been proposed; the transport electronic properties are investigated with the different geometry structures [44, 45]. The black–blue phosphorene lateral heterojunction has similar structure properties, as shown in Fig. 1c [46]. The black phosphorene and blue phosphorene composed of the same element have small lattice mismatch along the zigzag direction. There are two interfaces between them: octatomic-ring interface and hexatomic-ring interface. The octatomic-ring interface presents metallic features because of the in-gap state form of the interface and edges. The hexatomic-ring interface presents a metal-to-semiconductor transition feature with hydrogen passivation. By tuning the widths of the heterostructure component, the energy band can be modulated. The bandgap of N–N configuration heterojunctions (Fig. 1c, the black and blue phosphorene nanoribbons have the same width at 5:5) decreases with the increase in width N in the range of 1–10 nm. The electronic properties of N-5 configuration heterojunctions are similar to those of the N–N configuration heterojunctions. However, the electronic properties of the 5-N configuration heterojunctions are almost unchanged. Type-II and type-I band alignments can be observed in different width ranges. In the width ranges from 2.0 to 3.1 nm and 3.7 to 4.2 nm, type-II band alignment can be observed, because the conduction band minimum (CBM) state is mainly from the blue phosphorene and the valence band maximum (VBM) state is mainly from the black phosphorene. Outside of these width ranges, the band alignment is type I. When the width is very small, the CBM and VBM are mainly from the blue phosphorene; when the width is very large, the CBM and VBM are mainly located at the black phosphorene. With the strain on the heterostructure, the transition in type-II and type-I band alignments can also be observed. The newly found GeP_3_ 2D materials follow the rules of even–odd oscillating bandgap with a changeable armchair nanoribbon width. The lateral heterojunction has been designed with different armchair GeP_3_ nanoribbon widths (the width of one segment is even, and the width of the other segment is odd) [47]. The odd-width segment has a smaller bandgap than that of the even-width segment. It is found that the CBM charge density comes from the wider part, while the VBM comes from the narrow part. The heterojunction exhibits a type-II band alignment which can be easily tuned by the ribbon width and can potentially be used in future photoelectronics.

The periodic nanoholes on single-layer graphene can open its bandgap [48]. This kind of defect is considered as a powerful way to modulate electronic properties. The defects of different sizes, shapes, and periodicities of the nanohole in the lattice have been widely discussed [49, 50]. Bai et al. [51] fabricated a novel graphene nanostructure called graphene nanomesh (GNM), which can open up a bandgap in monolayer 2D graphene using block copolymer lithography methods. This work intrigued a set of investigations about GNM, especially on the electronic properties of graphene nanomesh [52, 53]. The nanohole on monolayer 2D materials is an effective way to tune the materials’ properties and form heterojunctions. The nanoholes on graphene can open an energy gap, which makes it become a semiconductor. The negative differential conductance (NDC) effect has been found in GNM *p*–*n* junctions, which proved the possibility of constructing a device with nanoholes without electrostatic tuning [54]. In Fig. 1d, a simple GNM heterostructure model, which has circuit holes, has been shown. With a similar structure, the mechanical properties of GNM heterojunctions have been researched with different shapes of nanoholes, including circular, square, and triangular holes [55]. Because the strength and ductility are reduced with the introduction of nanoholes, the heterojunction based on GNM may have limits in applications.

Furthermore, a single material heterojunction can also be modulated with the thickness. Because of the quantum confinement effect, the bandgap of monolayer MoS_2_ increases from indirect 1.29 eV of multilayer MoS_2_ to direct 1.85 eV. The changing layer numbers lead a new path to form heterojunctions, which are modified only by the thickness rather than by changing the composition. Monolayer and multilayer MoS_2_ heterostructures, which have type-I band alignments, have been reported [56]. Photocurrent can clearly be observed in the LHSs, which suggests potential application in future optoelectronic devices.

#### Doping and Passivation

**Fig. 2:**
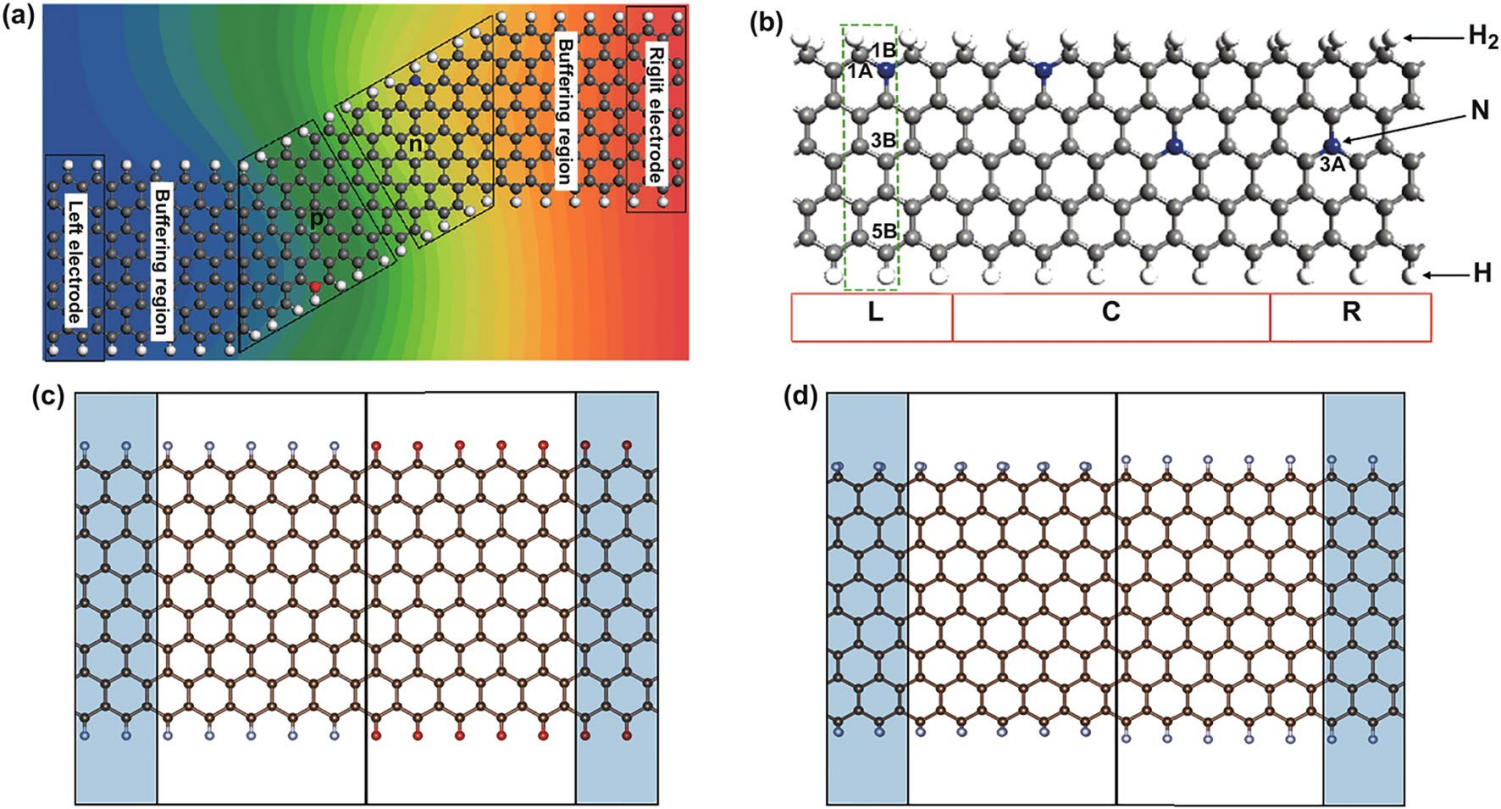
**a** Model of 10-armchair GNR *p*–*n* junction; the electrode is semi-infinite 14-zigzag GNR. The gray, white, blue, and red balls are C, H, N, and B atoms, respectively. Reprinted with permission from Ref. [62]. **b** Structure of H_2_–5zGNR–H. The blue, gray, and white balls represent N, C, and H atoms, respectively. L, C, and R denote the left electrode, scattering region, and right electrode, respectively. There are two unequal A and B sub-lattices in green rectangle. The atoms are labeled as 1B… 3B… 5B, and 1A is close to 1B; 3A is close to 3B; the sequence increases from top to bottom. Reprinted with permission from Ref. [75]. **c** Heterostructure of hydrogen (gray balls) and oxide (red balls) atoms passivated zGNR on the left and right side, respectively; the brown balls are carbon atoms. **d** Heterostructure of dihydrogenated zGNR (left side of the structure is passivated by two hydrogen atoms) and monohydrogenated zGNR (right side of the structure is passivated by one hydrogen atom). (Color figure online)

Doping is widely used in modulating the electronic properties of semiconductors and can also be used to design LHSs. *N*-doped and *P*-doped graphene nanoribbons have been investigated in detail. The graphene nanoribbons exhibit different electronic properties which depend on different deposition positions and deposition densities [57, 58]. *N*-doped and *B*-doped graphene nanoribbons have already been synthesized in experiments [59–61], which inspired the investigation of devices jointing the two differently doped graphene nanoribbons. As shown in Fig. 2a, the *N*- and *B*-doped m-aGNR junctions (where *m* represents the width of the aGNRs), which are tailored as a *Z*-type, have been designed, and the doping position has been kept relatively unchanged at the edge. The zGNR–aGNR atom interfaces have small contact resistance. The barrier is mainly attributed to the donor and acceptor doping on the aGNR. The different dopings on the semiconducting aGNR cause the rectification effect, which is related to the aGNR width. When the length of aGNR *p*–*n* junction is 3*n* and 3*n* + 2, positive rectification can be observed; when the length satisfied 3*n* + 1, negative rectification can be observed. The results suggest that the different rectification properties can be obtained by proper doping and adjusting the length of the junction [62]. Many *p*–*n* junctions by changing the doped position have been proposed, and the NDR or rectification phenomenon can be found there [63–71]. These devices can potentially be used in nanoelectronics and nano-random memory or molecular rectifier.

Zigzag graphene nanoribbons are usually used to design spin electronic devices because of their edge states [72, 73]. Wang et al. investigated zGNR heterojunctions, in which the zGNR electrodes are doped with N or B atoms and the edges are asymmetric. (One of the zGNRs edges has C–H bonds, while the other edge has C–H_2_ bonds.) The structure with asymmetry edge hydrogenation is stable and has good spin properties. There are two states close to the Fermi energy: The valence states belong to the up spin, while the conduction states belong to the down spin. It is suitable for spin device design. The doping on the GNRs, especially with B or N atoms, can also change the spin transport properties of GNRs. The doping atoms can create holes/electrons carriers, which modulate the spin transport properties. A bipolar spin filtering effect with a spin polarization of 100% can be observed in these doped zGNR-based heterojunctions [74]. In the analogous structure doped with nitrogen, the rectification of spin-polarized current can be tuned by changing the doping atom positions, as shown in Fig. 2b [75]. The chemical doping position can modify the electronic properties by breaking the symmetry of the structure, which may cause the rectification effect. A large rectification ratio (~ 10^5^) and a perfect spin filtering effect have been obtained with the structures. The boron nitride- and nitride-doped structures with asymmetry hydrogenation have also been studied to realize the spin filtering effect and rectification behavior [76]. These results are helpful in future spintronic devices designs.

For 2D graphene, the dangling bonds of edge atoms would be saturated by atoms or molecules to stabilize the edge structures. The edge modification of zGNRs can also influence the edge magnetism and reduce the edge state. Hydrogen atoms were mostly used to passivate the 1D nanoribbon [77]. Other atoms and different hydrogenation styles also have been adopted to modulate device electronic properties [78, 79]. As shown in Fig. 2c, the heterojunction is formed by passivating the left side with hydrogen atoms and the right side with oxide atoms. Heterojunctions with O atom-terminated zigzag graphene nanoribbon (O/zGNR) and H atom-terminated zigzag graphene nanoribbon (H/zGNR) have been discussed [80]. Different edge decoration atoms can modify the electronic properties of the GNRs and also the transport properties. In this case, the oxygen edge decoration nearly has no effect on current with positive bias but reduces current with negative bias. O/zGNR–H/zGNR heterojunctions show a clear rectification effect. Edge decoration atoms also influence the spin transport properties of the device. In this way, a remarkable dual spin filtering behavior has been observed on O–zGNR–H/H_2_–zGNR–H heterojunctions [81]. The spin degeneracy is lifted by the edge decoration. Different edge atomic groups can provide different band structures and produce different transport properties.

Heterojunctions with asymmetric edge hydrogenation (the left part of the structure saturated with one H atom, the right part saturated with two H atoms) have been fabricated, as shown in Fig. 2d. It is found that the C–H_2_ bonds showed an important influence on magnetic properties [82, 83]. Cao et al.[84] theoretically studied three kinds of heterojunctions: bare and dihydrogenated zGNR junction, bare and monohydrogenated zGNR junction, and dihydrogenated and monohydrogenated zGNR. (Bare zGNR is zGNR that is not saturated on both edges.) Their calculation results implied that the dihydrogenation edges lead to the blocking of electron transfer, which induced the rectification effect [72]. Therefore, it is clear that asymmetric edge hydrogenation can modulate electronic properties and form novel heterojunctions. Many similar heterojunctions have been fabricated this way. Zigzag-edged MoS_2_ nanoribbon passivated with one hydrogen atom (zMoS_2_NR–H) and zMoS_2_NR heterojunctions, which exhibit large NDR and rectification properties, have been designed; perfect spin filter effect reaches up to 95% spin polarization [85]. Zigzag-edged silicene nanoribbon (zSiNR) with different edge hydrogenation also shows some potential electronic and spin electronic applications [86]. H–6zSiNR–H/Ho–6zSiNR–Ho heterojunction exhibits perfect spin filtering effect with almost 100% spin polarization. H–6zSiNR–H/H_2_–6zSiNR–H_2_ heterojunction can exhibit dual spin filter effect and NDR phenomenon.

#### Strain and Dielectric Modulation

**Fig. 3:**
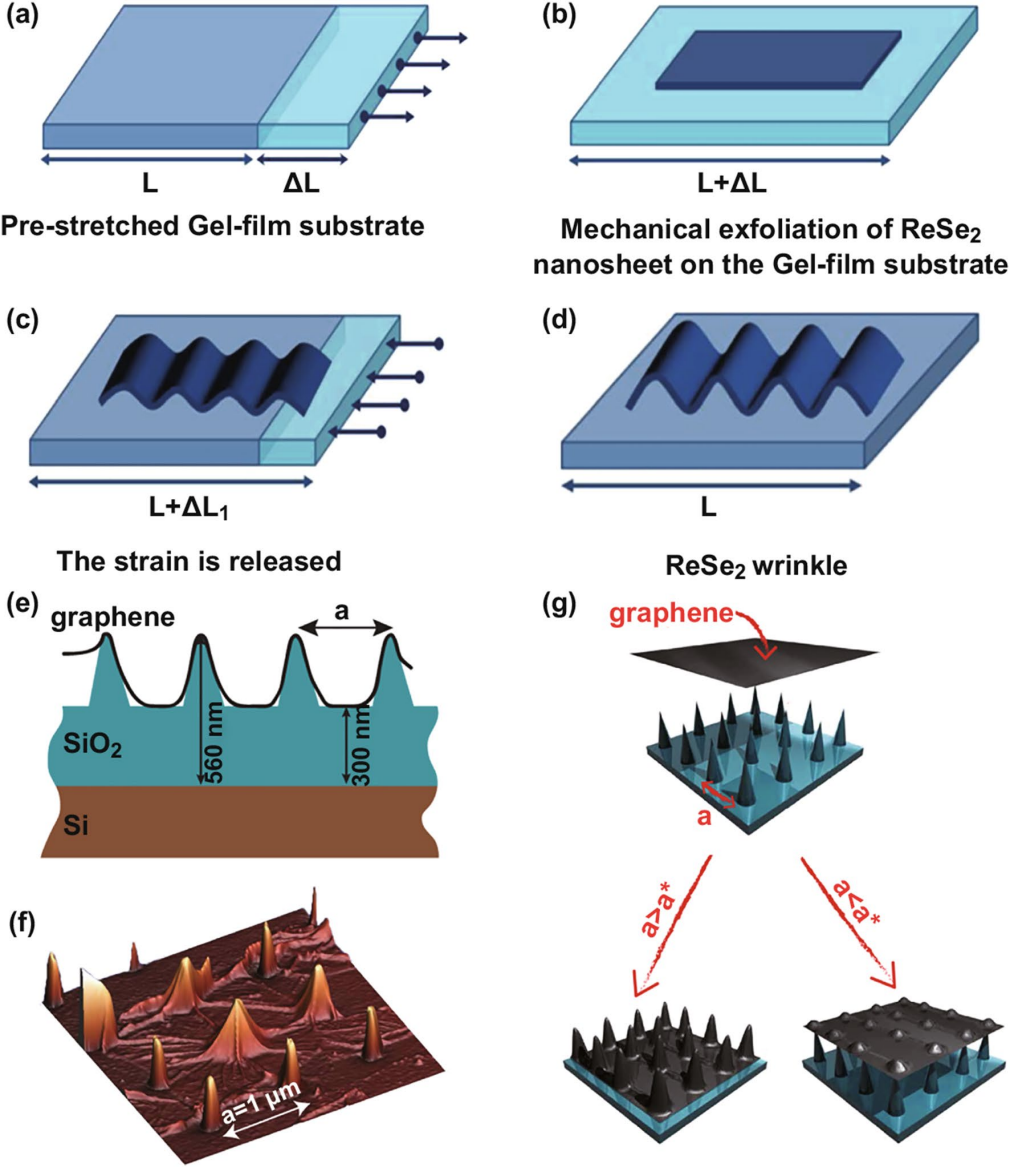
Schematic illustration of the fabrication steps of ReSe_2_ wrinkles. **a** Prepared the elastomeric gel–film substrate. **b** Deposited the monolayer ReSe_2_ with mechanical exfoliation method. **c** Released the strain with different rates. **d** Formation of ReSe_2_ wrinkles, which are perpendicular to the initial axis. Reprinted with permission from Ref. [87]. **e** Schematic diagram of graphene film, which is deposited on SiO_2_ nanopillars substrate. **f** AFM image of graphene membrane that deposits on the SiO_2_ nanopillars substrate. **g** Schematic diagram of transferring the graphene on the SiO_2_ nanopillars substrate, which has different density arrays. Reprinted with permission from Ref. [91]

Applied strain is another effective way to tune the electronic structures of 2D materials and has been used to realize strain-tuned superlattices, which are important members of LHJs [87–93]. When the applied strain is large (e.g., ~ 20% in graphene), the in-plane strong covalent interaction of the 2D materials makes it helpful in keeping their structures from bond-breaking. As demonstrated in Fig. 3a–d, a stretching-transfer-releasing process can induce designed strain on monolayer 2D materials. The large strain caused by the mismatch of elastic modulus between 2D monolayer and its substrate results in the formation of a periodic rippled structure. This structure behaves like a superlattice containing two different materials and shows a strain-modulated electronic structure. This strategy has been proved in the fabrication of strain-modulated superlattice in ReSe_2_ [87] and black phosphorus [88]. The local strain engineering can induce a controllable strain on the bandgap and then tailor the optoelectronic properties, which opens the door for a variety of applications including photovoltaics, quantum optics, and two-dimensional optoelectronic devices. Along this route, how to apply strain with different directions (tensile or compressive) and magnitudes is one of the key steps to modulate the bandgap. Besides this, the ability to apply a spatially controllable strain is even more crucial and desirable because graded bandgap semiconductors are eagerly needed in device designs. Designed dielectric nanopillar structures on a substrate, which sometimes are named as corrugated substrates, supply us more controllable degrees of freedom in tuning electronic structures of 2D materials and become a powerful synthesis route for strain superlattices in macroscale. According to this route, the nanopillars, which are made of a dielectric material, are placed between 2D monolayer materials and the substrate. The size and separation of the pillars control the strength and spatial pattern of the strain. The periodic pillars induce large strains with controllable patterns, as shown in Fig. 3e–g. The structures of the pillars provide the strain-dependent tenability of the electronic structures and strain-modulated graphene superlattice [89, 91–93]; MoS_2_ superlattice [90] has been fabricated successfully using this method. By inducing designed strain distributions, optical properties of 2D materials show a great improvement. For example, it is shown experimentally that strain-engineered MoS_2_ has a broad band light absorption with absorption bandwidth from 677 (unstrained MoS_2_) to 905 nm (most strained MoS_2_), which covers the entire visible wavelength and most intensive wavelengths of the solar spectrum [90].

As summary, through the two strategies mentioned above, nonuniform strain field and related considerable energy gap variation can be controllably realized, which enables strain-engineered 2D materials to be potentially applied to optoelectronic device designs. Furthermore, pseudomagnetic fields provided by the engineered nonuniform strain interact with electrons and induce changes in the bandgap, which show very interesting physical phenomena and have aroused studies on pseudomagnetic fields in 2D materials [94].

Under a strain-engineering mechanism with corrugated substrates, the structure of nanopillars (size and separation) controls the strength and spatial pattern of the strain. However, the complex morphology makes the interpretation of stress more difficult especially when the pitch and sharpness of the corrugated array are taken into account. It is an interesting question on whether other substrate properties show modulation on the electronic properties of the above 2D materials. A recent work confirmed this with their theoretical and experimental investigations on a dielectric-defined lateral heterojunction in a monolayer semiconductor MoS_2_ [95]. The bandgap renormalization due to the dielectric screening effect by substrate(s) is dramatic, especially when the 2D materials have a low intrinsic dielectric constant. In this work, a continuous monolayer MoS_2_ was prepared on a substrate with a high dielectric constant (*ε*) and an adjacent segment on a substrate with a low *ε*. Because of the dielectric constant-dependent renormalization of the electronic bandgap, the bandgaps of MoS_2_ introduced by the two substrates on each segment show a difference, and the monolayer MoS_2_ forms an in-plane type-I heterojunction above the boundary of the two substrates. This type-I heterojunction generated by the dielectric constant-induced bandgap renormalization in atomic 2D materials will have profound effects on their electrical transport properties. Both the structure and dielectric distribution can be used to tune physical properties of 2D materials and construct lateral heterojunctions.

### Heterogeneous Junctions

LHSs based on one material are monotonous. However, heterostructures based on different materials have been designed and studied a lot, with a variety of 2D materials. Vertical heterostructures based on heterogeneous materials have been widely investigated by changing the rotations of different stacking layers [96, 97], or the stacking components in consideration of the great quality of the 2D materials [98, 99], or the layer thickness [100], or the interlayer spacing [101–103], or the stacking mode [104–106]. However, while the tunable mechanisms of vertical structures based on different materials have been discussed a lot, the tunable mechanisms of LHSs with different materials are of less concern. Here, we discuss mainly the tunable mechanisms of LHSs based on two different materials. When LHSs are formed with different materials, the band alignment is important in determining the electronic and optical properties. Hence, the LHSs are classified as metal/semiconductor and semiconductor/semiconductor contacts, as follows.

#### Heterogeneous Junctions with Semiconductor/Semiconductor Contact

**Fig. 4:**
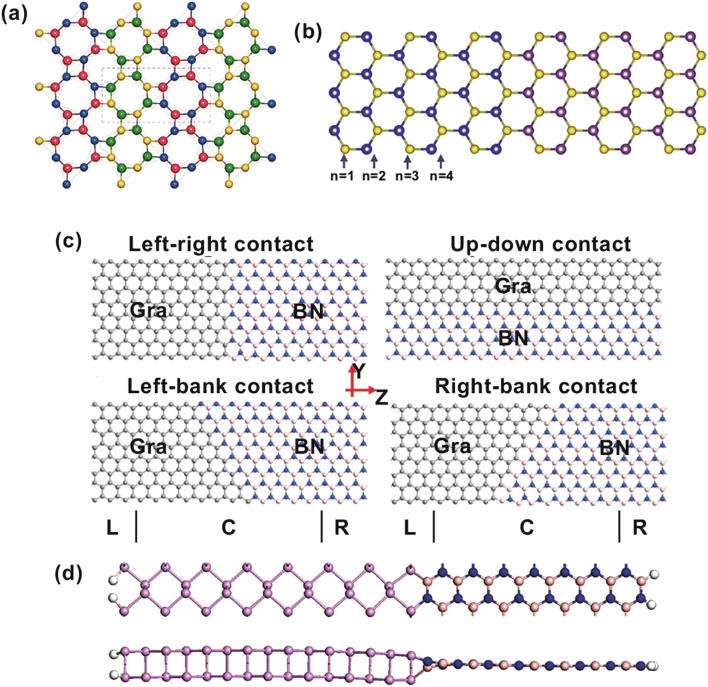
**a** Atomic structure of 1S–MX_2_ (*M* = Mo/W, *X* = S/Se) LHS from the top views. Yellow: S, indigo: Se, red: Mo, green balls: W. Reprinted with permission from Ref. [107]. **b** Top view of MoS_2_/(MX_2_)n LHSs, where MX_2_ represents MoSe_2_, WS_2_, or WSe_2_; n refers to the width of the substitution region. Reprinted with permission from Ref. [108]. **c** Atomic structures of lateral graphene/BN heterostructures, which have different contact types. Reprinted with permission from Ref. [116]. **d** Top and side view of the heterostructure of 15-aPNR/15-aGNR (aPNR is armchair phosphorene nanoribbon; aGNR is armchair graphene nanoribbon). Reprinted with permission from Ref. [118]. (Color figure online)

Lateral TMDCs 2D heterostructures have been researched a lot because of their unique electronic properties, especially potential type-II band alignment and the wide distribution from semimetal to semiconductor. The electronic properties and inner mechanisms of more and more TMDCs LHSs have been discussed. The 2D square TMDCs (1S–MX_2_) (*M* = Mo, W, *X* = S/Se) with metallic properties were reported [different from the conventional phase of 2D MX_2_ (named 1H–MX_2_)], and having a similar structure to that of 1H–MX_2_, a 1S–WS_2_ and 1S–MoSe_2_ lateral monolayer heterostructure has been built, as shown in Fig. 4a. The wanted type-II band alignment can be observed with black phosphorus as substrate. The theoretical researches on 1S–MX_2_ LHSs provide opportunities in new device physics and optical application [107]. MoS_2_–(MX_2_)_*n*_ (*M* = Mo/W, *X* = S/Se) LHSs have been studied with first-principal calculations, as shown in Fig. 4b [108]. The band levels and optical absorption of the structures are influenced mainly by the width n of the MX_2_ region, where MX_2_ represents MoSe_2_, WS_2_, or WSe_2_. The CBM states and VBM states of MoS_2_-(WS_2_)_*n*_ lateral heterostructures are contributed mainly by the MoS_2_ region and (WS_2_)_*n*_ region, respectively. This property is related to the width n. When the width *n* > 2, the MoS_2_–(MX_2_)_*n*_ lateral heterostructures show type-II band alignment. The free electrons and holes are separated in the MoS_2_ and MX_2_ regions, respectively, which induce the long electrons’ and holes’ lifetimes. The width-tuned heterostructure provides desired electronic, optical, and photocatalytic properties. The 2D WS_2_/WSe_2_/MoS_2_ in-plane structures are also discussed [109]. The bandgap of the heterostructures can be tuned by adjusting the lengths of the components, which are induced mainly by the confinement effects. When the width increases from 1 to 5 (unit cell), the bandgap decreases.

In addition, the strain can modify the electronic structures and band offsets. With the strain-tuned transition of type-I and type-II band alignment, LHSs have potential application in light-emitting and photovoltaics. Uniaxial tensile strain can modulate the electronic and optical properties of TMDCs LHSs, MoX_2_–WX_2_ (*X* = S, Se, Te). With the bandgaps and band offset tuned by the uniaxial tensile strain, the light conversion efficiency of the structure can be elevated [110].

The interface of heterogeneous junctions is the current research focus. The lateral interfaces, both zigzag and armchair, of monolayer TMDCs, such as MoSe_2_–WSe_2_ and MoS_2_–WS_2_, have been investigated. The interface of LHSs plays an important role in device properties. It is found that these interfaces behave as 1D state with noticeable features [111]. Of course, heterogeneous junctions can combine different kinds of materials, which provide opportunities to tune novel electronic properties. Some TMDCs materials of less concern, such as lateral monolayer ZrS_2_ and HfS_2_ nanoribbons, have also been used to fabricate heterostructures [112]. The AlN–GaN nanoribbons heterostructure has been investigated [113]. The stability of AlN–GaN nanoribbons increased when the GaN ratio increased. The bandgap can vary inversely when the GaN content increased. All the nanoribbons show semiconductor behavior with indirect bandgap. An arsenene/blue phosphorene LHS is formed from a zigzag arsenene monolayer and a zigzag blue phosphorene monolayer [114]. Its bandgap can be tuned by the component ratio of the structure and tensile strain along different directions. Given the same component ratio, the bandgaps will decrease when the widths of the heterostructures increase. While the width increases, the VBM of the heterojunction shifts upward and the CBM shifts downward at the г point. As_2_P_2_ LHSs behave as quasi-type-II indirect semiconductors. As_*m*_P_*n*_ (*m* = *n* = 4, 6, 8, 10) LHSs behave as type-II direct semiconductors, with the VBM localized around arsenene and the CBM localized around blue phosphorene. The electronic properties can change from indirect bandgap to direct bandgap as the width increases. Heterostructures of different component ratios have been discussed. For heterostructures of As_*m*_P_20-*m*_ (*m* = 2, 4, 6, 8 …18), when *m* < 10, the CBM shifts downward while the VBM shifts upward. When *m* > 10, the CBM shifts upward while the VBM shifts downward. With increasing width, the bandgap first decreases and then increases. The tensile strain will not change the direct bandgap property. However, the strain has a strong effect on the CBM and VBM states. When the applied strains exceed 6%, the band alignment transition from type II to type I can be observed. The tunable heterostructures have type-II band alignment and high carrier mobility, which can potentially be used in photovoltaic, optoelectronic, and photocatalytic devices.

#### Heterogeneous Junctions with Metal/Semiconductor Contact

Gapless graphene can easily form M/S contact with other 2D materials, such as BN, phosphorene, and TMDCs. The electronic properties of LHSs are highly related to the M/S interface. Lateral graphene/BN heterostructures with different kinds of interfaces, armchair or zigzag, have been studied [115]. The intrinsic strength is highly related to the misorientation angle of the interface, and the interface following Clar’s rule has higher tensile strengths. Under uniaxial strain, the bandgap of the heterostructure with zigzag interface is nearly unchanged, but the bandgap with the armchair interface is changed. The theoretical results show the interface effect on the mechanical and electronic properties, which can give guidance to the design of lateral hybrid heterostructures. In-plane graphene and h-BN heterostructures with different contacts have been investigated, as shown in Fig. 4c [116]. When the interface is left–right-type contact, rectification phenomenon can be observed. The up–down contact heterostructure clearly exhibits the NDR effect. When the contact is left bank or right bank, it displays the NDR effect and a large rectification ratio. With change in the contact structure, different NDR and rectification properties can be obtained. To get knowledge on the growth mechanism of graphene–h-BN heterostructures, the growth pathways, including the influence of Cu substrate, electronic properties that proved to be metallic, and chemical bonds of graphene/BN LHS, have also been discussed based on DFT theory [117]. The work promotes the understanding of the evolution of the characteristics of the graphene/BN heterostructures’ growth. Because of a small mismatch of phosphorene and graphene along the armchair direction, in-plane phosphorene/graphene heterostructures(aPNR/aGNR) can be built, as shown in Fig. 4d [118]. The theoretical results imply that the electronic properties, such as bandgaps of the heterostructure, can be tuned by the widths of GNR and PNR. Furthermore, the doping hydrogen in the heterostructures can reduce the bandgap and induce transition from semiconductor to metal. A two-probe graphene/phosphorene/graphene device has been designed, which behaves with tunneling transport characteristics.

The electronic transport properties of four different edge contacts (armchair–armchair, armchair–zigzag, zigzag–zigzag, zigzag–armchair) between graphene and MoS_2_ have been studied [119]. The MoS_2_ become more metallic because of the gap state from the interface. The difference potential of the four contact geometries implies that the interface plays an important role in carrier transport of graphene/MoS_2_ junctions. The structure preference with C–S or C–Mo on growth condition, the charge transfer, and the mid-gap states of the boundary in the lateral graphene–MoS_2_ interface have also been discussed [120]. These properties are helpful in future electronic device design.

## Applications of LHSs: Electronic and Photoelectronic Devices

### Electronic Devices

**Fig. 5:**
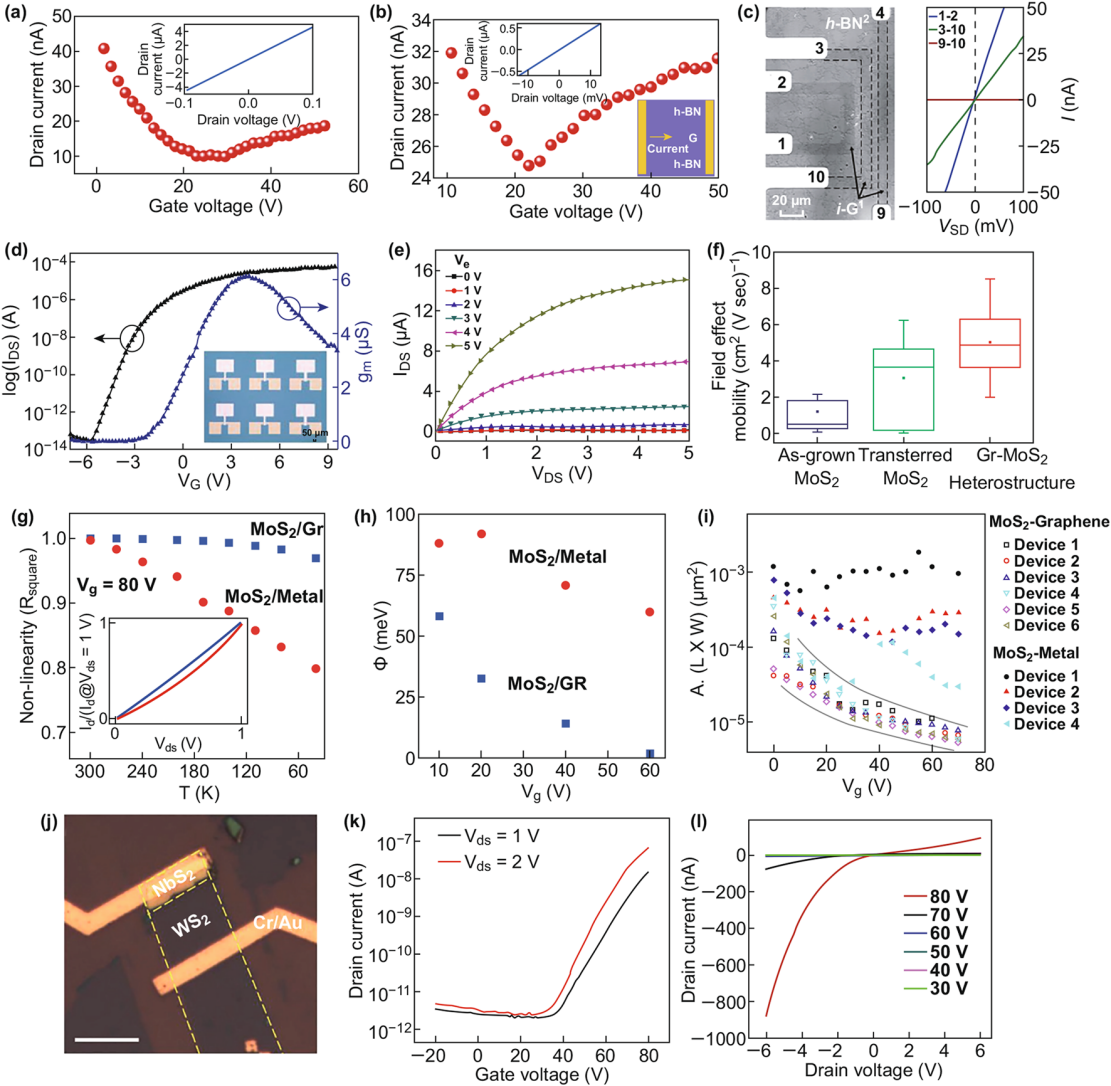
Electrical measurement of graphene/h-BN FET with current flowing **a** along the stripes and **b** perpendicular to the stripes (inset: *C*–*V* curves and diagram of the device). Reprinted with permission from Ref. [124]. **c** Optical image of graphene/h-BN heterostructure with electrodes on the graphene (indicated by the dash lines) and two-terminal current–voltage curves of different devices. Reprinted with permission from Ref. [126]. **d** Transfer and transconductance (*g*_m_) plots of graphene–MoS_2_ FETs at *V*_ds_ = 1 V. **e** Output plots of graphene–MoS_2_ FETs at gate voltage ranging from 0 to 5 V. **f** Field-effect mobility of different MoS_2_ FETs, including as-grown, transferred MoS_2_, and graphene–MoS_2_ FETs. Reprinted with permission from Ref. [121]. **g** Linear regression (*R*_square_) in *I*_d_–*V*_ds_ at different temperatures (normalized with the *I*_d_ at *V*_ds_ = 1 V). **h** Measured Schottky barrier of MoS_2_–graphene and MoS_2_–metal FETs. **i** Noise amplitude of MoS_2_–graphene and MoS_2_–metal FETs at different *V*_g_. Reprinted with permission from Ref. [123]. **j** Optical image of NbS_2_–WS_2_ FET device. **k** Transport property of NbS_2_–WS_2_ FET. **l** Output plots of NbS_2_–WS_2_ FET at different backward voltages. Reprinted with permission from Ref. [122]

Electronic devices based on lateral heterostructures, such as FETs [121–123], resonators [124], and logic circuits [125], which exhibit unique device properties, have been fabricated. In FET devices based on graphene/h-BN heterostructure film, the drain current, which is along and perpendicular to the heterostructure, has been measured, as shown in Fig. 5a, b [124]. The inset depicts the linear *I*–*V* curves obtained in zero gate, which implies tunable transport properties under different biases. The calculated mobilities of the two devices are ~ 1700 and 520 cm^2^ v^−1^ s^−1^, respectively, which exhibit good field-effect mobility. Moreover, such devices have good plane integration properties. With this kind of heterostructure, flat electrically isolated graphene devices can be fabricated [126]. Monolayer h-BN has a sheet resistance larger than 400 TΩ. Two-terminal devices and their *I*–*V* characteristics are shown in Fig. 5c. Graphene contact devices show conducting behavior, and h-BN contact device shows insulator behaviors. The electrically isolated graphene devices can be used in ultra-flat three-dimensional electronics.

Lateral graphene–TMDC heterostructures exhibit some good electronic properties. Top-gate FETs with graphene as source and drain and with MoS_2_ as channel, have an on/off ratio of ~ 10^9^ and maximum transconductance g_*m*_ of ~ 6 µs [121]. The transfer and transconductance (*g*_m_) curves are shown in Fig. 5d, and the inset depicts the schematic diagram of the FETs. The output curves in the gate voltages range of 0–5 V are shown in Fig. 5e. The mobility of this FET is also higher than that of an as-grown and transferred MoS_2_, which is shown in Fig. 5f. Compared with the MoS_2_–metal heterostructures FETs, the MoS_2_–graphene FETs have a lower Schottky barrier [123]. Linear regression (*R*_square_) in *I*_d_–*V*_ds_ at different temperatures is plotted in Fig. 5g. To get knowledge on the linearity of the *I*_d_–*V*_ds_ trends in MoS_2_–graphene and MoS_2_–metal FETs, the inset is plotted at a temperature of 270 K. Compared with MoS_2_–graphene FETs, MoS_2_–metal FETs have larger nonlinearity in the *I*_d_–*V*_ds_ curves, which means the presence of a larger barrier. The Schottky barrier extracted from Arrhenius measurements in different *V*_g_ shows that the MoS_2_–metal heterostructures FETs have a larger barrier, as shown in Fig. 5h. In Fig. 5i, it is shown that the MoS_2_–graphene heterostructures also have lower noise than the MoS_2_–metal devices, because of the lower barrier in the contact.

Based on TMDC–TMDC heterostructures, NbS_2_ and WS_2_ FETs have been built [122]. The triangular NbS_2_ and WS_2_ lateral heterostructure is etched as a ribbon, and then Cr/Au is deposited on the ribbon as electrons. The optical image is shown in Fig. 5j. The transfer characteristic curves of this device are shown in Fig. 5k, which show an *n*-type behavior. The device has a large on–off ratio of 10^5^. The output characteristic (*I*_ds_–*V*_ds_) is shown in Fig. 5l. The *I*_ds_ decreases with the *V*_ds_ from 80 to 30 V. From the output characteristic, a current rectification behavior can be observed. Although the calculated field-effect mobility is 0.14 cm^2^ V^−1^ s^−1^, the electronic properties can be improved in the future. The devices are hopeful future integrated electric devices and integrated circuit applications.

### Optoelectronic Devices

**Fig. 6:**
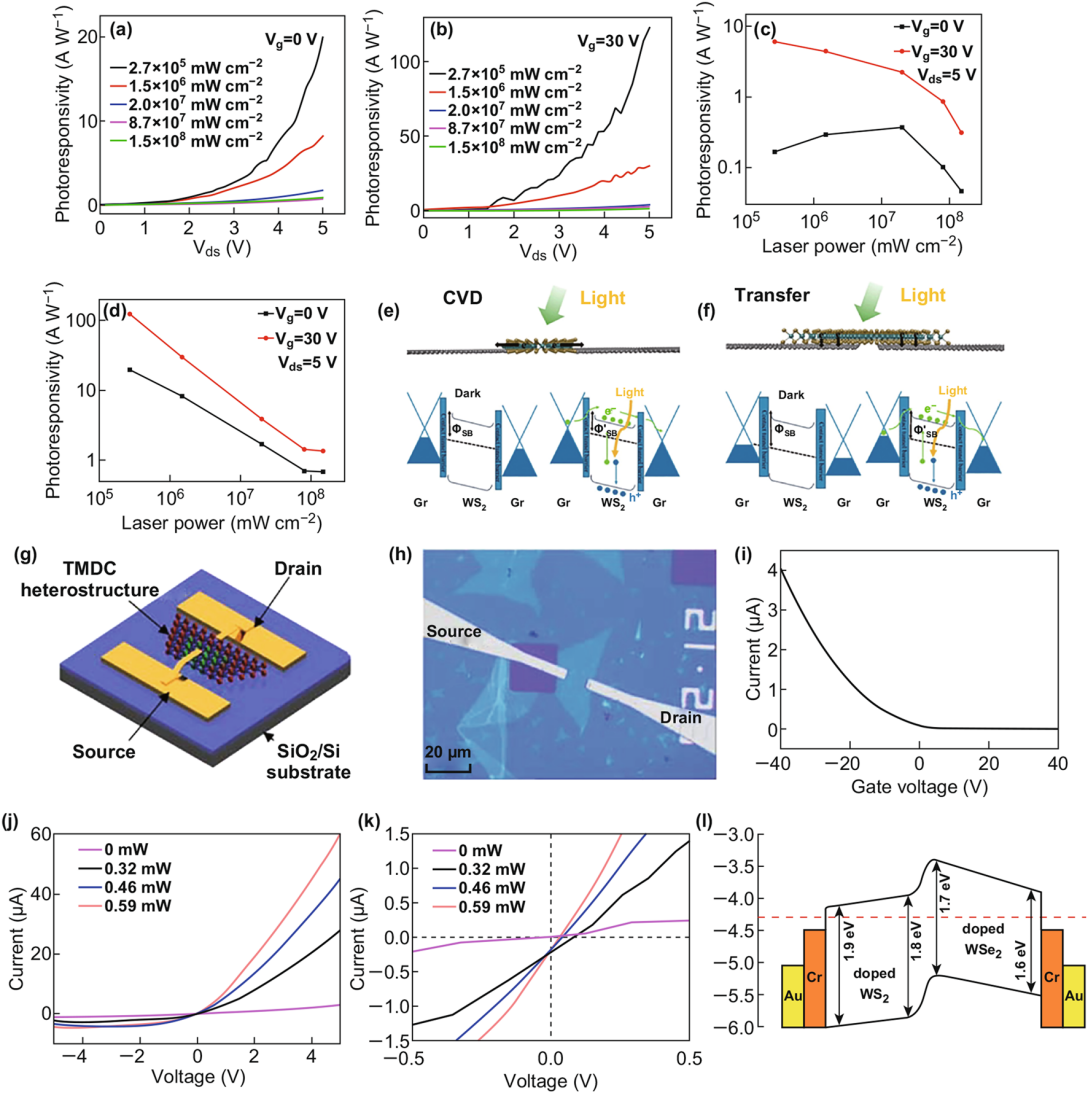
Photoresponsivity of graphene–WS_2_–graphene under **a** 0 V, **b** 30 V gate voltage. Photoresponsivity of FET formed by **c** transferring, and **d** CVD method. **e**, **f** Devices and band diagram of graphene–WS_2_–graphene FETs made by transfer and CVD method. Reprinted with permission from Ref. [127]. **g** Diagram of photodiode built with WSe_2_–WS_2_ heterostructure. **h** Optical image of the photodiode in **g**. **i** Drain current of the WSe_2_–WS_2_ photodiode without light illumination. **j** Transport curves (*I*_ds_–*V*_ds_) under different illumination powers (*V*_g_ = 0). **k** Transport curves in voltage ranging from − 0.5 to 0.5 V. **l** Band alignment of the WSe_2_–WS_2_ heterostructure with zero bias. Reprinted with permission from Ref. [130]

The optoelectronic properties of lateral heterostructure devices have been investigated intensively. Some optoelectronic devices exhibit superior optical performance [127–130]. In lateral graphene–TMDC heterostructures, photons excite electron–hole pairs from the TMDC and rapidly transfer the electrons to the graphene in the Schottky junction, which provides a good photoresponse property. The lateral graphene–WSe_2_–graphene photodetecting transistors exhibit photoresponsivity reaching up to 121 A W^−1^ under 2.7 × 10^5^ mW cm^−2^ illumination, as shown in Fig. 6a, b; the photoresponsivity of on (*V*_g_ = 30 V) and off (*V*_g_ = 0 V) states is given under different laser powers [127]. In Fig. 6c, d, the photoresponsivities of the heterostructure with two different structures are measured in the same experimental conditions, and the lateral heterostructures synthesized with CVD method have better photoresponsivities than those of the transferred heterostructure. The superior photoresponsivities of the devices are mainly attributed to the low barrier in the graphene and WSe_2_ contact. The band diagram of graphene–WSe_2_–graphene heterostructures is depicted in Fig. 6e, f. The structure grown with CVD method has smaller contact resistance and lower barrier than the transferred structure, which promote electron transfer and increase photoresponsivity. With lateral graphene–MoS_2_ heterostructures, the photodetector has a specific detectivity D* of up to 1.4 × 10^14^, which is of importance in future applications [129].

Because of their desirable type-II band alignment, TMDC–TMDC LHSs have attracted a lot of attention. The type-II band alignment promotes the separation of electrons and holes, leads to the long lifetime of photoexcited charge carriers, and reduces the recombination of electrons and holes at the surface. Heterostructures with type-II band alignment is suitable for optoelectronic device design. The schematic and optical image of a lateral doped WSe_2_–WS_2_ heterojunction photodiode [130] built by CVD method is shown in Fig. 6g, h. The drain current of the device changing with gate voltage in Fig. 6i without illumination indicates the *p*-type behavior of the materials. The electronic transfer curves under different illumination powers ranging from 0 to 0.59 mW in Fig. 6j, k show that the photocurrent becomes larger when the illumination power increases. In Fig. 6l, because of the type-II band alignment diagram at the zero bias, the electrons prefer to migrate from WSe_2_ to WS_2_. A forward bias can easily shift the electrons, but a reverse bias cannot always shift the electrons in the reverse direction. The highest photoresponsivity of this device is 6.5 A W^−1^ in a light illumination of 0.32 µW at a wavelength of 532 nm. The external quantum efficiency (EQE) of this heterostructure can also reach up to 15.2%. Above results confirm that WSe_2_–WS_2_ heterostructures are proper elements for high-performance optoelectronic devices.

### Brief Summary

In order to obtain a brief insight on LHSs, we list typical LHSs in Table 2. In this table, we summarized the main properties of the LHSs, including structures, properties, remarkable parameters and performances, synthesis, and applicable devices.

**Table 2:** Summary of the LHSs

LHSs	Properties	Parameter and performance	Synthesis	Devices	Refs.
aGNR|zGNR	Rectification	Max rectification ratio (MRR) ~ 10^4^	–	*p*–*n* diode	[41]
armchair(m)/armchair(n) GNR	Rectification	Rectification ratio > 1	Bottom up	*p*–*n* diode, molecular switches	[43, 132]
GNM/graphene	A high peak current, peak-to-valley ratio	PVR ~ a few hundred	–	*p*–*n* diode	[54]
*n*-doped/*p*-doped GNR and *n*-doped GNR/GNR (*n*-doped GNR)	Rectification and NDR	MRR ~ 2 × 10^6^	–	NDR molecular device, molecular rectifier	[63, 64, 67, 126, 131]
		MPVR ~ 10^5^, mobility > 10,000 cm^−2^ V^−1^ s^−1^	Bottom up, two-step growth		
H_2_-doped (m)zGNR–H	Bipolar spin filtering effect, NDR, and rectification	The spin polarization reach 100%, MRR ~ 10^5^	–	Spin filter	[75]
O/zGNR–H/zGNR	Rectification	MRR = 9.93 × 10^8^	–	Rectifier	[80]
H_2_–(m)zGNR–H/H–(n)zGNR–H	Rectification, dual spin filtering effect	MRR ~ 10^5^, spin polarization reach 100%	–	Spin rectifier, spin filter, Magnetoelectronics device	[72, 83]
H_2_–zGNR–H/H–(doped)zGNR–H	Dual spin filtering effect, NDR	Reach 100% spin filtering efficiency	–	Spin filter	[76]
zMoS_2_NR–H/zMoS_2_NR	Spin filtering effect, negative differential resistance, rectification effect	Reach 95% spin polarization, MRR ~ 67	–	Spin filter	[85]
H–6ZSiNR–H/H_2_–6ZSiNR–H_2_	Dual spin filter and NDR, spin rectification effect	Spin polarization ~ 100%, MRR ~ 48	–	Spin filter	[86]
Graphene–h-BN	Rectification and NDR	Mobility ~ 190–2000 cm^−2^ V^−1^ s^−1^, MRR ~ 9	One-step growth, two-step growth	Field-effect transistors, split closed-loop resonator, thin integrated circuitry	[116, 124, 126]
Graphene–MX_2_	Fermi level pinning	Mobility ~ 11.5 cm^−2^ V^−1^ s^−1^, responsivity ~ 121 A/W, detectivity ~ 1.2 × 10^13^ Jones, on–off ratio ~ 10^9^	Two-step growth	Photodetectors, field-effect transistors, logic devices	[121, 127, 128]
Graphene–aPNR	Quantum size effects, tunneling transport characteristics	–	–	–	[118]
h-BN–MX_2_	–	–	Two-step growth	–	[142]
(MX_2_)*m*–(MX_2_)*n*	Light-emitting and photovoltaic, photocatalysis, photoelectronic	On–off ratio ~ 10^6^, responsivity ~ 6.5A/W, detectivity ~ 2.6 × 10^11^ Jones, internal quantum efficiency ~ 91%, incident photon conversion efficiency ~ 0.12%, open-circuit voltage ~ 0.47 eV, short-circuit current ~ 1.2 nA	One-step growth, two-step growth, multi-step growth, laser, strain, thermal, plasma treatments, BPE (bipolar electrode deposition)	Transistors, CMOS inverters, photodetectors, photodiode, light-emitting devices	[21, 22, 130]

GNM is graphene nanomesh, H_2_ refers to the edge passivated by two H atoms, H refers to the edge passivated by one atom, O refers to oxygen atoms, MX_2_ represents the 2D TMDCs materials, aPNR refers to armchair phosphorene nanoribbon, MRR is the max rectification ratio, MPVR is the max peak-to-valley current ratio

## The Experimental Synthesis of LHSs

The excellent properties of the LHSs discussed above inspire more works about the synthesis of LHSs. It is expected that the synthesis of LHSs can promote more device applications. In this section, we review the experimental synthesis of the LHSs.

Although the previous proposed LHS devices have great performance, their applications are hindered by the synthesis technology. Recently, with the developments in synthesis technology, many LHS devices, such as graphene heterostructures and graphene devices, have been successfully fabricated. Graphene nanoribbon heterojunctions were synthesized with a bottom-up method in 2014. These devices behave in a similar way to traditional p-n junctions [131]. Moreover, width-modulated armchair graphene nanoribbon heterojunctions have also been synthesized with a bottom-up method [132]. In LHS synthesis, CVD methods are the most widely used and effective ways. These CVD methods can be classified as one-step, two-step, and multi-step growths according to the process steps. One-step growth is a self-assembled process where it is difficult to control the shape and size of the interface. The two-step and multi-step methods move forward in solving these difficulties by introducing additional process steps. Different LHSs have been synthesized through one-step growth, two-step, and multi-step growths and some other methods. We summarized the synthesis methods for LHSs as follows.

### CVD Synthesis Route I: One-Step Growth

The lateral graphene–h-BN heterostructure was synthesized. In 2010, an h-BN and graphene heterostructure was synthesized using a CVD method, which randomly distributed h-BN and graphene domains in the monolayer hybrid structure. The hybrid structure has a different bandgap compared to those of h-BN and graphene [133]. Fabricated h-BN and graphene LHSs with one-step growth are scarce; nonetheless, TMDC LHS fabrication with this method has developed a lot. TMDCs, especially MoS_2_ and WSe_2_, are extremely popular 2D materials which were used to build 2D heterostructures. These structures usually have type-II band alignment, which is desirable and can be used to build potential electronic and optoelectronic devices [134]. The synthesis of TMDC heterostructures had drawn the interest of many researchers and was developed very fast. In 2014, Duan et al. built WS_2_–WSe_2_ and MoS_2_–MoSe_2_ LHSs laterally through an epitaxial process with the CVD synthesis method. The built heterostructure has a gradual and seamless interface and exhibits good electronic and optical properties [21]. Gong et al. reported a one-step synthesis method for the creation of both vertical and lateral WS_2_–MoS_2_ heterostructures with a vapor-phase growth process. The monolayer WS_2_ vertically grown on the MoS_2_ forms a bilayer heterostructure in high temperature, and the WS_2_ laterally grown on the edge of MoS_2_ forms an in-plane heterostructure in a lower temperature [135]. The lateral structure has a clean and atomically sharp interface. The LHS can serve as an intrinsic p-n diode without external gating. A one-step atmospheric pressure chemical vapor deposition (APCVD) strategy is proposed to synthesize a lateral WS_2_–MoS_2_ heterostructure, which is simplified and low cost. The built heterostructure has a high-quality, sharp subnanometer interface. The built-in potential of the heterostructure has been characterized, which is significant in future photoelectrical applications [136]. The 2D WS_2_/Mo_1−*x*_W_*x*_S_2_/WS_2_ LHSs with a concise one-step CVD method on Si substrates have been achieved, by which the formation of different structure components of the LHSs was tuned by the growth temperature and chronology [137]. With the synthesis technology development of 2D heterostructures, time–temperature–architecture (TTA) diagrams were proposed to describe the synthesis of MX_2_ (*M* = Mo, W; *X* = Se, S) heterostructures with the single-step CVD method [138]. With control over the CVD process, the different structures, such as lateral, vertical, or hybrid, and alloys, can be synthesized, respectively. The time and temperature are the main factors to control the process. The established TTA framework generalizes the one-step CVD process and complemented the works mainly on vertical heterostructures and alloys, also concerned with LHS synthesis. This diagram may promote applications of the synthesis technique.

**Fig. 7:**
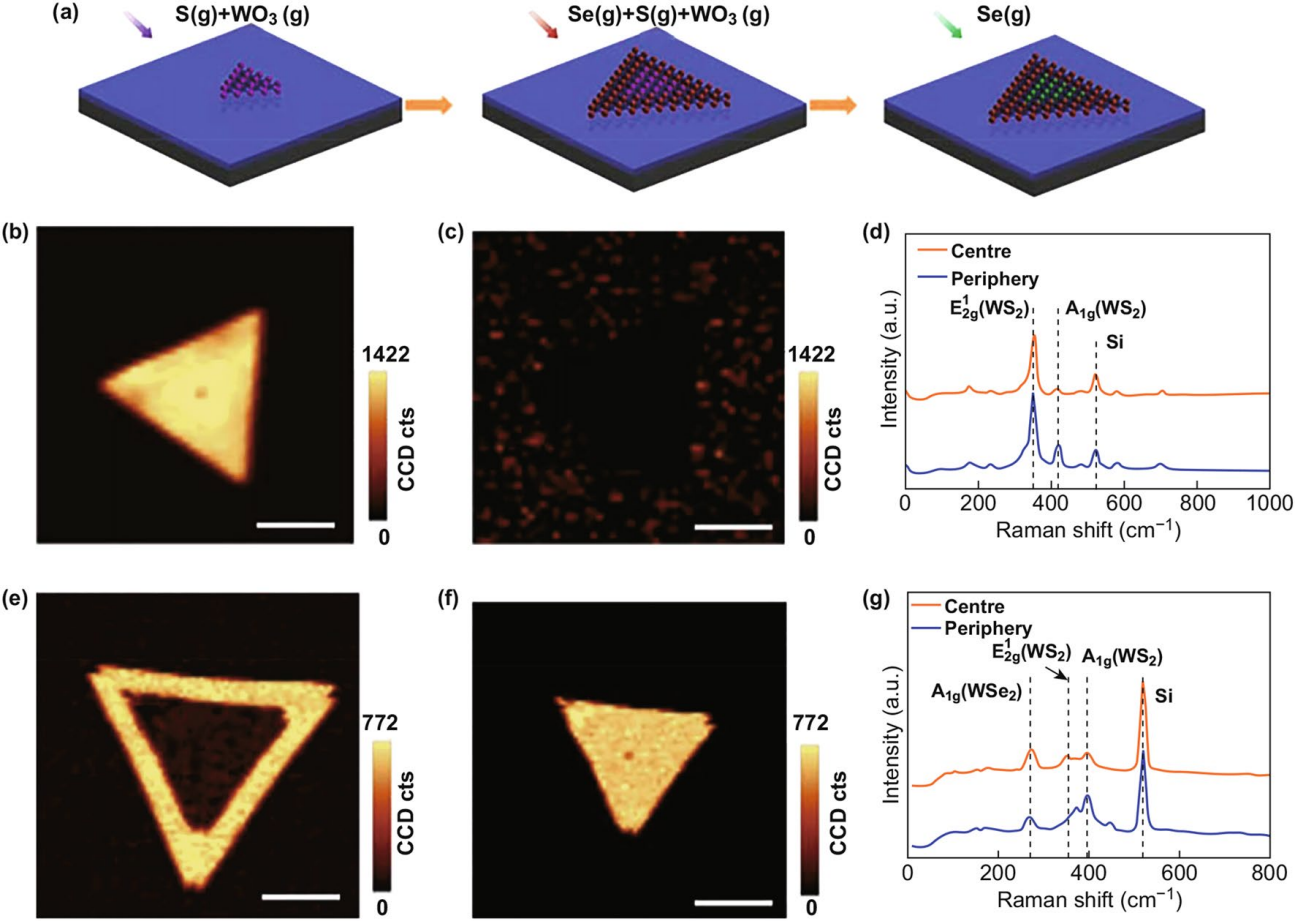
**a** Growth processes of doped WSe_2_–WS_2_ LHS. Raman image of the sample in **b–d** second and **e–g** third process. Reprinted with permission from Ref. [130]

Graded doped lateral WSe_2_–WS_2_ heterostructure has been fabricated using the one-step growth technique in ambient pressure [130]. The one-step CVD method only has one heating cycle, showing merits of simplicity and cheapness. The elemental substitution enables graded composition distribution and provides a tunable bandgap, which reduces the large band bending at the interface. The one-step growth can be divided into three processes, as shown in Fig. 7a. The monolayer WS_2_ is grown first, followed by the epitaxial growth of WS_2_ doped with Se, and then, the central part is converted into WSe_2_ because of the overloaded Se. To confirm this, the second and third processes are further observed in an experiment. In the second process, WS_2_ epitaxial growth is achieved with overloaded S. The Raman image in Fig. 7b depicts the structure of WS_2_ in the center region. Se-doped WS_2_ can be observed at the edge from Fig. 7c. The nonexistent peak of WSe_2_ in the Raman shift in Fig. 7d confirms that epitaxial growth happened in this process. After processing in the furnace for the third step, S-doped WSe_2_ is clearly shown in Fig. 7e, f in the central region. The appearing WSe_2_ peak in the Raman spectra in Fig. 7g indicates the formation of WSe_2_. With this method, graded doped lateral heterostructures can be synthesized through the above-mentioned three sequential processes.

### CVD Synthesis Route II: Two-Step Growth

Because the mentioned one-step growth techniques are self-assembled, it is difficult to control the shape and size of the heterostructures. In this section, we discuss mainly the two-step growth process based on CVD method, including metal/semiconductor contact (graphene–h-BN, graphene–TMDC) and semiconductor/semiconductor contact (TMDC–TMDC) LHSs, in which most researchers are interested.

#### The Two-Step Growth of M/S LHSs

In 2012, hybrid graphene and hexagonal boron nitride sheets have been synthesized with a two-step CVD method. The graphene was grown from Cu foil, and then, the h-BN grew from the graphene grains on the bare Cu foils [139]. The h-BN/graphene heterostructures with spatial control were fabricated [126], which was combined with photolithography and etching techniques. The graphene layer was first grown with the CVD method, then patterned with photolithography, and had the unwanted areas etched, followed by the selective growth of the h-BN layer. The LHSs have high carrier mobility and plane integration, which are suitable for integrated circuit fabrication. An in-plane monolayer graphene and h-BN heterostructure, which has controlled shape, sharp interface, and large size, has been built with similar synthesis methods [124]. The LHSs have nice portability, which can be easily transferred to other platforms. The sequence of growing such a structure is different from the previous one; the h-BN film is grown first with the CVD method and had some h-BN lithographically etched, followed by the growth of graphene on the etched area. Furthermore, a graphene and h-BN heterostructure with a straight-line interface has been demonstrated along the graphene crystallographic orientation with an APCVD method [140]. The graphene is grown on the Cu foil, followed by the h-BN grown from the graphene template. The sharpness of the interface can be controlled by the conditions during growth. A zigzag boundary graphene and h-BN heterostructure has been achieved with a two-step CVD method on Cu foil; the zigzag boundaries were formed with a hydrogen etch, and the h-BN can keep the lattice orientation of graphene [141].

**Fig. 8:**
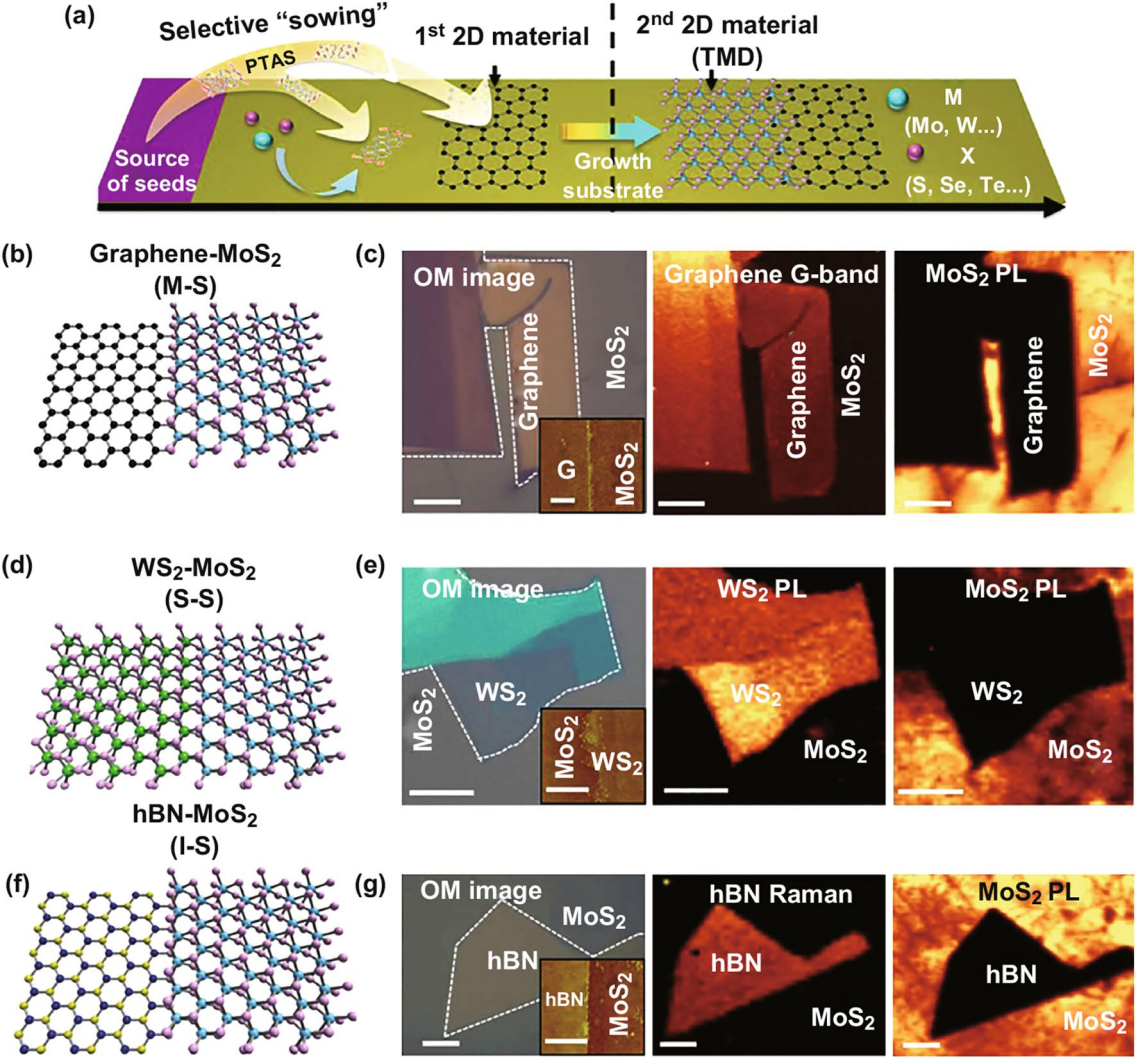
**a** Schematic diagram of the synthesis of parallel-stitched 2D-TMDC heterostructure. **b**, **d**, **f** Schematic diagrams of graphene–MoS_2_, WS_2_–MoS_2_, and h-BN–MoS_2_. **c**, **e**, **g** Optical image and spectroscopy intensity mapping images of graphene–MoS_2_, WS_2_–MoS_2_, and h-BN–MoS_2_. Reprinted with permission from Ref. [142]

In 2016, lateral graphene–MoS_2_ heterostructure synthesized with aromatic molecules as the seed has been reported [142]. The process is illustrated simply in Fig. 8a. The aromatic molecules are used to control the reaction speed. The monolayer graphene was first transferred on the substrate as seed, followed by the aromatic molecules sowed on the nearby bare substrate which made a hydrophilic surface; the second layer MoS_2_ can grow rapidly along the first layer. The schematic diagram, optical image, and spectroscopy intensity mapping image of graphene–MoS_2_ heterostructure are given in Fig. 8b, c. This method can also provide a route for other 2D building blocks and plane-integrated device designs. Later, the lateral MoS_2_–graphene heterostructure is fabricated with seed-free direct growth method. The graphene flake is transformed on SiO_2_/Si substrate, and then, the MoS_2_ grows along the graphene with atmospheric pressure CVD method. The device based on it has high mobility and low noise, which has better performance than MoS_2_–metal devices [123]. Large-scale and high-quality graphene and WS_2_ LHS have been fabricated without using seeding. The graphene is synthesized first and then lithographically etched by oxygen plasma. The WS_2_ is selectively grown on the etched area, forming a film. The LHS has an ohmic contact between graphene and *n*-doped WS_2_ [143]. A graphene/MoS_2_/graphene LHS has been fabricated with a similar two-step method. The MoS_2_ was grown between two stripes of graphene on Cu foil. The photodetector based on it has a quick photoresponse and specific detectivity [128].

#### The Two-Step Growth of S/S LHSs

With the one-step process, it is difficult to grow p-n heterostructures like WSe_2_–MoS_2_, in which both the metal and chalcogen elements are changed at the same time. The two-step growth methods can overcome these difficulties. In 2015, a lateral WSe_2_–MoS_2_ heterostructure was synthesized with the two-step lateral epitaxial growth method. The process avoided alloy formation and formed an atomically sharp interface. Although there is a large lattice mismatch, the method provides a reliable means to produce monolayer components for future monolayer electronics [144]. Almost at the same time, Gong et al. demonstrated a two-step CVD method for growing TMDCs heterostructures: The MoSe_2_ was grown first and then the WSe_2_ epitaxy grew on the edge and on top of the MoSe_2_ layer. With control over the growth time, different types of heterostructures can be formed. Because the size of each 2D component can be controlled, the two-step methods can grow large heterostructures with sizes of up to 169 µm, and the cross-contamination can be reduced, compared with the one-step growth method [145]. Chen et al. used a simplified two-step CVD technique to fabricate an in-plane MoS_2_–WS_2_ heterostructure with a facile growth process in ambient pressure. The lateral heterojunctions behave as intrinsic *p*–*n* diodes and clearly exhibit the photovoltaic effect. The technique can potentially be applied to the growth of TMDC superlattices [146].

The method with aromatic molecules as seeds, which can accelerate growth, can also be used to synthesize TMDC–TMDC and h-BN–TDMC lateral heterostructures [142], as shown in Fig. 8a. This general method has been successfully applied in fabricating the graphene–TMDC heterostructure, which is mentioned in the previous section. This method is expected to have a large-scale production capability. With this method, the 2D materials and TMDC parallel-stitched heterostructure can be formed, without consideration for the lattice mismatch. The schematic diagram of WS_2_–MoS_2_ and h-BN–MoS_2_ heterostructures, and the optical image and spectroscopy intensity mapping image of these two heterostructures are depicted in Fig. 8d, f and e, g, respectively. The boundary of the heterostructure is marked with the white dash lines, and the heterojunction structure is confirmed with an optical image of the AFM in the inset picture, which shows the clear interface of the heterojunction. A large-area, high-quality, mosaic MoS_2_–MoSe_2_ LHS was synthesized with a two-step CVD method. In this method, the triangle monolayer MoS_2_ is synthesized first, and then, the MoSe_2_ is grown along the MoS_2_ edges, filling the black of the substrate, which forms a clear interface. The photodetector based on the heterojunction film exhibits perfect photoresponse performance. The method can provide a route for other mosaic lateral heterojunction films, which may have optimal photoelectric properties [22]. A novel two-step growth method with and without ion exchange has been demonstrated. If the MoS_2_ grows first in 730 °C, then the WSe_2_ grows at 875 °C, the selenium atoms replace the sulfur atoms, and the WSe_2_ and MoSe_2_ heterostructure are formed. If the growth is in reverse order, the WSe_2_ grows at 875 °C first and then the MoS_2_ grows in 730 °C, and the WSe_2_ and MoS_2_ heterostructure can be formed without ion exchange. Using this method, the monolayer MoS_2_ and WSe_2_ domain sizes can grow up to 100 µm on SiO_2_/Si substrates [147].

### CVD Synthesis Route III: Multi-step Growth

**Fig. 9:**
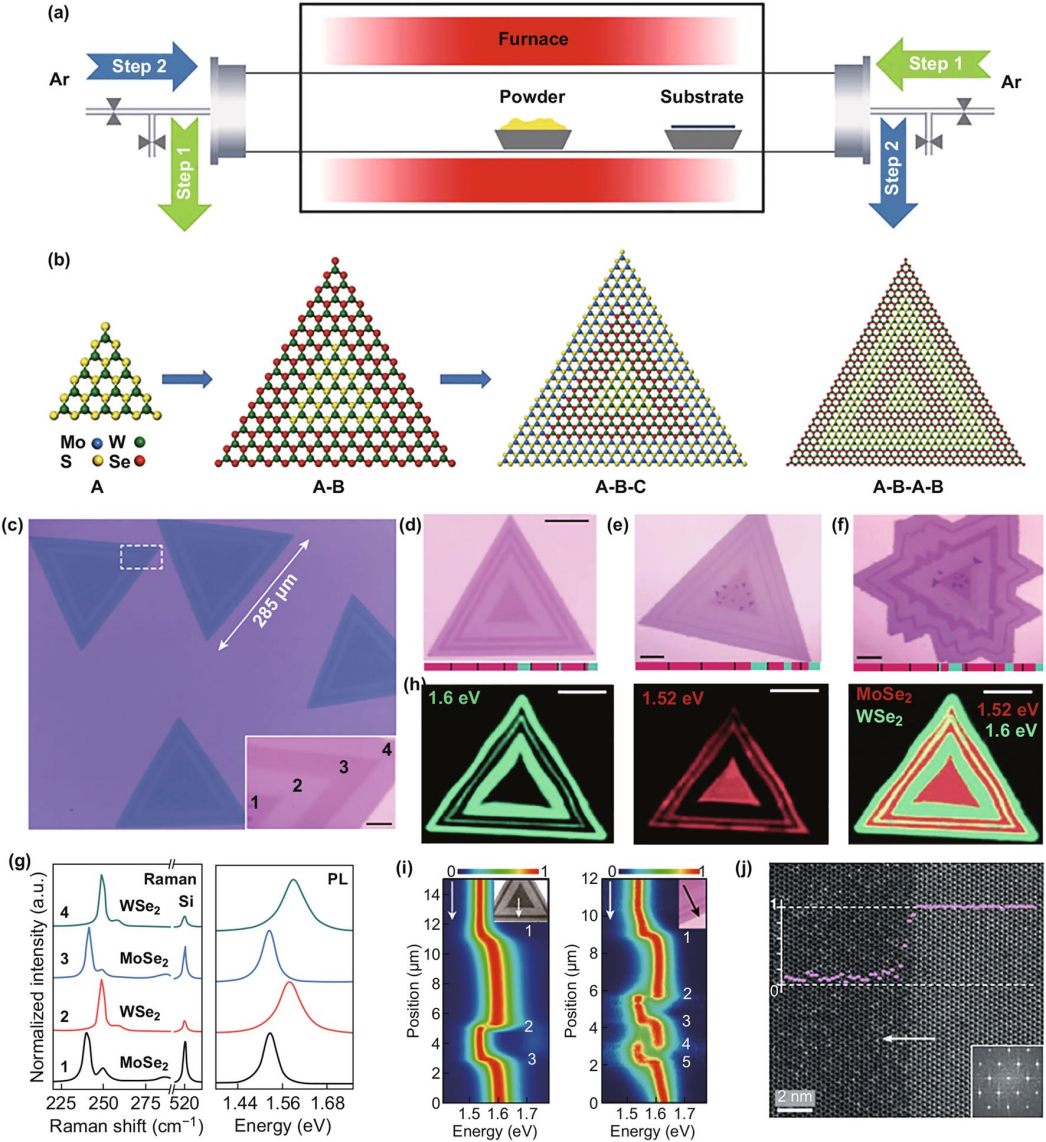
**a** Schematic diagram of multi-step CVD system for the growth of lateral heterostructures. **b** Schematic diagram of monolayer seed A, A–B heterostructure, A–B–C multi-heterostructure, and A–B–A–B superlattice. Reprinted with permission from Ref. [148]. Optical image of **c** three-junction and **d**, **e** five-junction MoSe_2_–WSe_2_ heterostructure; the thickness of MoSe_2_ can be distinguished with different colors. **f** Optical image of seven-junction MoSe_2_–WSe_2_ heterostructure with different domain widths. **g** Raman and photoluminescence spectra of positions 1–4 in **c**. **h** PL intensity maps for the heterojunction in **d**. **i** Contour color plots of the normalized PL intensity of three and five junctions. **j** HAADF-STEM image of the sharp interface of MoSe_2_–WSe_2_ heterojunction. Reprinted with permission from Ref. [149]. (Color figure online)

With the development of the one-step and two-step growth methods, many lateral heterostructures, which grow the second materials at the edge of the first materials, have been synthesized. However, it is still difficult to synthesize lateral heterostructures with multiple distinct materials blocks, which need continuous growth steps [148]. In the CVD process, the sequential growth cannot tolerate thermal-induced degradation. In addition, it is hard to control the chemical vapor sources in different temperature stages, which may lead to unwanted homogeneous nucleation. Therefore, the synthesized monolayer must endure the temperature and chemical environment swing in multiple steps. The unwanted homogeneous nucleation must be minimized. A step-by-step thermal CVD process has been designed, as shown in Fig. 9a. The source power is heated in a flow of argon carrier gas for each step, and the epitaxial layer is grown at the edge of the last monolayer crystal. To overcome the problem of excessive thermal degradation and uncontrolled nucleation in the multiple steps, a reverse flow is used to flush the existing monolayer materials on the substrate during the temperature swing. The forward flow is only applied at the exact growth temperature. With precise control over each step, the heterostructure, multi-junction heterostructure, and superlattices can be fabricated, as shown in Fig. 9b. Moreover, the built multi-junction heterostructure and superlattice have sharp interfaces, which provide desirable electronic properties. This method also provides a path to complex heterostructure synthesis with controlled location and orientation.

A one-pot synthesis method provides a solution to synthesize lateral multi-junction TMDC heterostructures [149]. MoSe_2_–WSe_2_ multi-junction heterostructures have been synthesized by controlling the gas-switching cycles. The number of junctions is determined by the cycles, and the domain size is controlled by the growth time of each cycle. The optical image in Fig. 9c indicates the synthesis of lateral three-junction MoSe_2_–WSe_2_ heterostructure, which has a size of up to 285 µm. As shown in the inset of Fig. 9c, the darker region is MoSe_2_ and the brighter region is WSe_2_. The optical images of five-junction heterostructure with different thicknesses are shown in Fig. 9d, e. The optical image of seven-junction heterostructure with different domain widths is depicted in Fig. 9f. The Raman and photoluminescence spectra can further confirm the composition distribution of the heterojunctions. The measured Raman spectra and photoluminescence (PL) spectra of positions 1, 2, 3, and 4 in Fig. 9c are demonstrated in Fig. 9g. The phone modes of positions 1 and 3 correspond to MoSe_2_, and the phone modes of positions 2 and 4 correspond to WSe_2_. The spatial distribution of the lateral heterojunction can also be confirmed with the Raman spectra. According to the PL spectra, there is a peak at around 1.52 eV for MoSe_2_ in regions 1 and 3 and a peak at around 1.6 eV for WSe_2_ in regions 2 and 4. The photoluminescence intensity maps for MoSe_2_ in 1.52 eV and for WSe_2_ in 1.6 eV are depicted in Fig. 9h. The composite photoluminescence map in the right panel of Fig. 9h exhibits the alternating concentric triangle domains. The contour plots of the normalized photoluminescence can characterize the interface quality; the three junctions and five junctions along the arrows in the inset are shown in Fig. 9i. From the left panel in Fig. 9i, it can be seen that the MoSe_2_ peak at 1.53 eV gradually shifts to the WSe_2_ peak at 1.6 eV in the first junction (labeled as ‘1’ in the figure). Nevertheless, the junctions in positions 2 and 3 have an abrupt shift, which indicates a sharp interface with fewer alloys. The sharp interface and high-quality crystal are confirmed in Fig. 9j with high-angle annular dark-field scanning transmission electron microscopy (HAADF-STEM). With the above methods, the high-quality multi-junction heterostructure and superlattice can be fabricated.

The CVD method can also be adopted to fabricate thickness-modulated LHSs. Zhang et al. formed bilayer–monolayer (BL–ML) thickness terraces with zigzag orientation lateral heterojunction with the CVD method, with which the second layer TMDC was grown on the monolayer TMDC [150]. The layer-modulated gap processed the type-I band alignment, which is different to the other CVD-method-synthesized type-II band alignment. He et al. [151] used the CVD method and obtained the different layers of MoSe_2_ junctions with uniform and smooth domain boundaries, which depend mainly on the control of temperature. It is a wonderful example on building layer-controlled large-scale 2D materials and heterojunctions.

### Synthesis Methods Other Than CVD

**Fig. 10:**
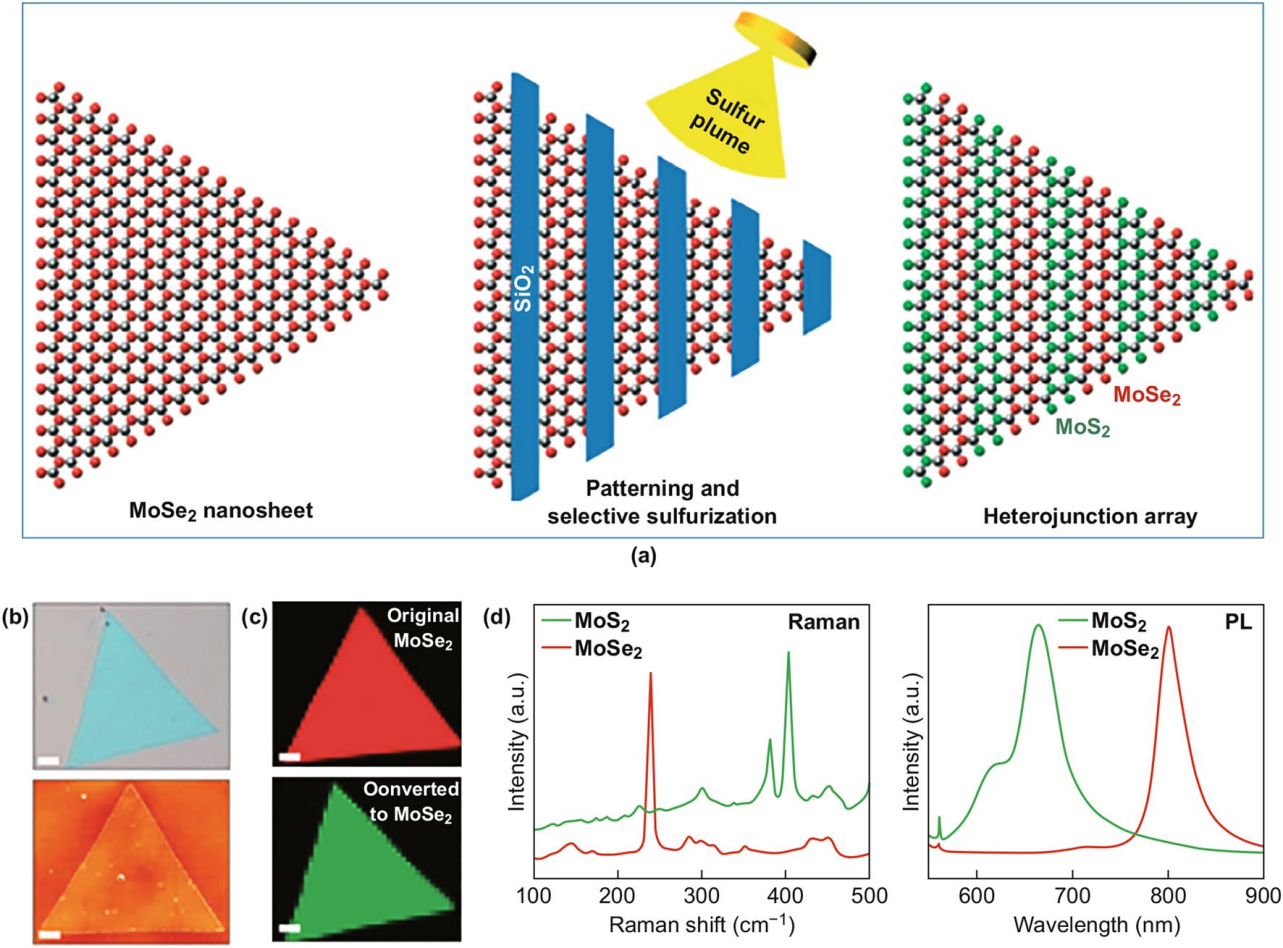
**a** Illustration of the steps for the formation of MoSe_2_–MoS_2_ heterojunction. **b** Optical and AFM images of MoSe_2_ in the size of ~ 40 μm. **c** Raman maps of the nanosheet before and after the conversion process (400 pulses at 700 °C). **d** Raman and PL spectra of original MoSe_2_ and converted MoS_2_. Reprinted with permission from Ref. [152]

**Fig. 11:**
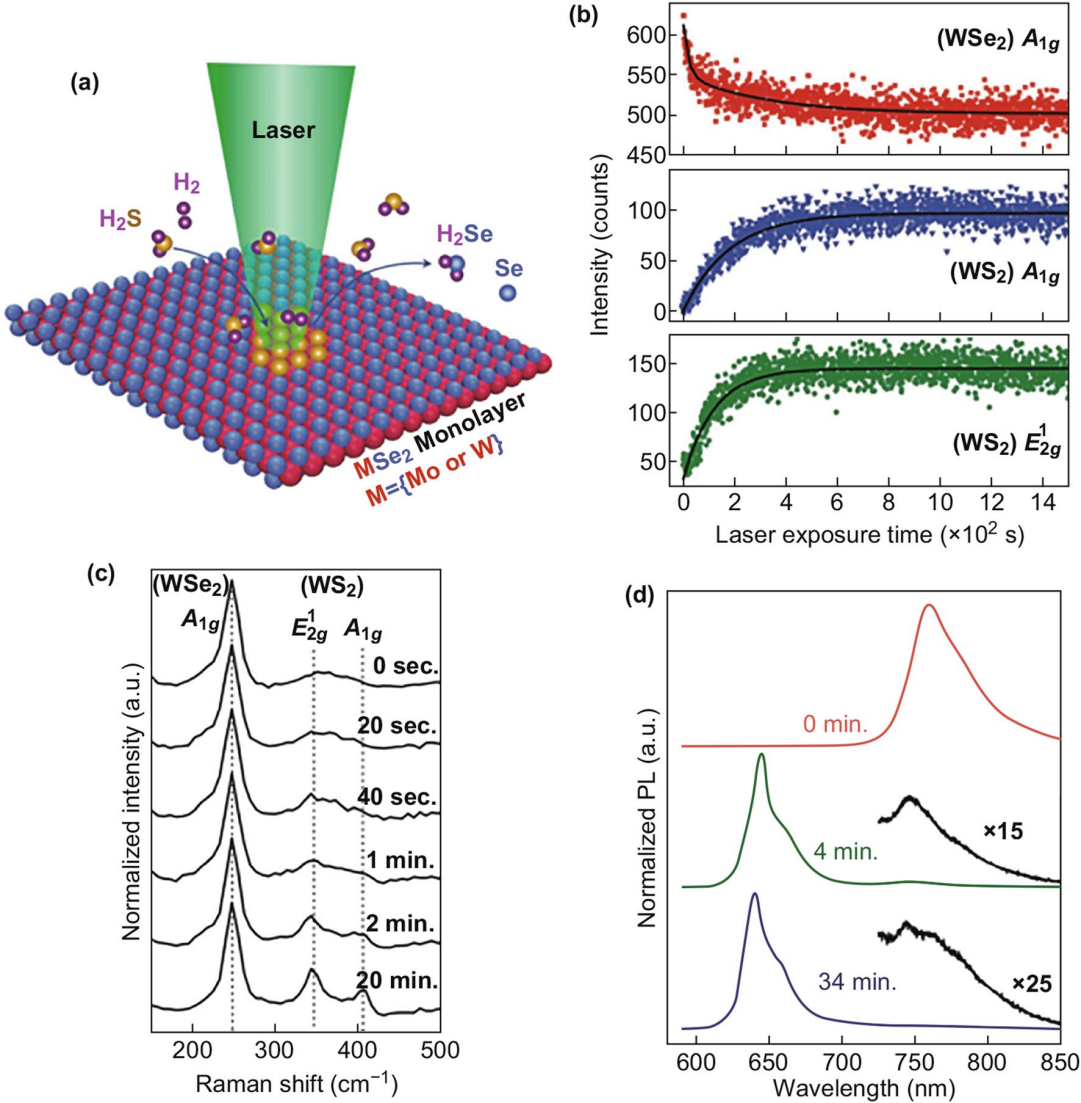
**a** Schematics illustration of laser-induced chalcogen atom exchange in H_2_S environment. **b** Raman intensity of WSe_2_ and WS_2_ under laser irradiation (523 nm, 0.7 mW) in H_2_S environment. **c** Raman spectra in different exposure times. **d** PL spectra in different times. Reprinted with permission from Ref. [153]

Many other methods have been proposed for the synthesis of LHSs. A lateral MoSe_2_–MoS_2_ heterojunction has been fabricated with electron beam lithography [152], as shown in Fig. 10a: The monolayer MoSe_2_ was first synthesized with CVD method followed by the deposition of SiO_2_ as a mask, lithographically patterned with e-beam, and had the uncovered part converted into MoS_2_ by pulsed laser vaporization of sulfur. To totally complete the conversion, the substrate temperature of MoSe_2_ should be higher than 600 °C, and the laser-vaporized sulfur pulses should be higher than 300. The total conversion is proved by the Raman and PL spectroscopy. The optical and atomic force microscopy images of monolayer MoSe_2_ are shown in Fig. 10b. The contrast of Raman maps between original MoSe_2_ and converted MoS_2_ is shown in Fig. 10c. The Raman and PL spectra of monolayer region before and after the conversion process are shown in Fig. 10d. It can be seen that the MoSe_2_ completely converted to MoS_2_ with this method and also lateral heterostructures and array can be formed, which can provide a sharp heterojunction interface (~ 5 nm) with easy spatial control. Different from the conversion method using sulfur plume, the laser-assisted method has been proven to be an effective way to modify the TMDCs, which can replace selenium with sulfur atoms [153], as shown in Fig. 11a. The photoconversion process happens in a controlled reactive gas environment. The spatially localized photoconversion process can form a TMDC heterostructure. The experimental conversion has been realized in suspended WSe_2_ and MoSe_2_ monolayer, with laser-induced modification in H_2_S environment. The selenide atoms can change to sulfide atoms in the process with the assistance of a laser whose power is in the range of 0.3–1 mW. The Raman peak intensities of WSe_2_ in H_2_S change with exposure time in Fig. 11b: The A_1g_ mode of WSe_2_ decreases over time, suggesting the reduction in W–Se chemical bonds; on the contrary, the A_1g_ and $E_{{2{\text{g}}}}^{1}$ of WS_2_ increase with time. It is suggested that the sulfur atoms increase in the lattices with exposure time. The Raman and PL spectra for different exposure times in Fig. 11c, d show that the chemical conversion from WSe_2_ to WS_2_ happened with the laser exposure time. This method provides an effective way to construct in-plane lateral heterojunctions.

Strain as an effective way to modify the physical properties of materials is a hopeful technique to realize the LHSs. The strain engineering of materials has been proposed for a long time. The 2D graphene has superior mechanical property, which can sustain elastic deformation of more than 25%. The wrinkles fabricated by applying uniaxial strain lead to additional damping on the graphene. The wrinkles on ReSe_2_ also have been achieved by introducing local strain [87] in the process shown in Fig. 3a–d. The monolayer ReSe_2_ is obtained by mechanical exfoliation method from ReSe_2_ crystal. Then, it is deposited on elastomeric substrates, which are prestretched by 30–100%. The wrinkles on ReSe_2_ can be formed by releasing the prestrain of elastomeric substrates. The defect tuned by the stress can provide a way to generate electrical gap and tune the optical properties.

The surface morphology of the substrate can also induce strain on the 2D materials. For example, the strain ripples have been generated by transferring the graphene membrane on the substrate which is corrugated by an array of SiO_2_ nanopillars [91], as shown in Fig. 3e. The different patterns of strain can be tuned by adjusting the pillar array geometry (size and separation) of the substrate. The atomic force micrograph of graphene deposited on SiO_2_ nanopillars in Fig. 3f provides geometry information on nanopillar arrays. The schematic steps of transferring graphene onto the nanopillar arrays are shown in Fig. 3g. The graphene is synthesized by the CVD method on the copper foil. Then, the SiO_2_ substrate is etched into nanopillars. The graphene is deposited on the substrate after acid-etching the copper foil under the graphene. Different nanopillar arrays induce different strain distributions in graphene. With the high-density arrays (*a* < a*), the graphene is suspended between the nanopillars. With the low-density arrays (*a* > a*), the graphene is fitted on the substrate, with highly symmetric rippers. Depending on the array geometry and pitch, graphene film can conform on the substrate, partially collapse, fakir-like, or suspend. The different configurations of strain domain will provide tunability for the electronic properties.

Through CVD, thermal [154] and plasma treatments [155] have been used to realize LHSs. The methods need multi-step fabrication processes and can apply only on specific materials. A simple micromechanical exfoliation technique is also used to form a mono–multilayer MoS_2_ type-I heterojunction [56]. This structure can exhibit some good photoelectric properties. Along this way, the lateral junction modulated by the thickness can be fabricated. Using bipolar electrodeposition (BPE) technique, Jamilpanah et al. [156] built a lateral heterostructure with type-I and II band alignments. Because of the renewal of the BPE technique in materials science, a one-step method including a quick growth of gradients of molybdenum sulfide and oxides along a conductive substrate was proposed. The experiment is processed at room temperature and requires only cheap experimental equipment. All these methods provide feasible means to conduct the synthesis of 2D lateral heterostructures, which provide potential prospects in future nanoscale devices.

## Perspectives

In this review, we summarized the physical properties of LHSs that can be tuned by the structures (interface, width, nanohole, and thickness), doping, passivation, strain, and dielectric. The device applications and experimental synthesis of LHSs have also been discussed. One-step, two-step, and multi-step growths based on CVD and other growth techniques have been used in the synthesis of heterogeneous junctions. Although there are a large number of works about LHSs, there are still a lot of unsolved issues. For example, in view of the tunable mechanisms, the doping- and passivation-induced heterogeneous junctions are seldom investigated, although the doping and passivation have worked as effective tuning methods in homogeneous junctions. Among the fields of LHSs, researchers pay most attention to the differences in materials in the heterogeneous junctions but neglect the geometrical effects on the properties of heterogeneous junctions. The strain or dielectric modulation has been used to construct homogeneous junctions, but there are very few works published so far. There is a lot of work that needs to be done. In view of the synthesis, the biggest challenge is to develop synthesis technology to fabricate homogeneous junctions where structures, doping, and passivation are precisely controlled. Studies of LHSs offer opportunities to design novel electronic, spin, and optical devices with desirable high performance.
